# Characterization of Industrial Lithium Iron Phosphate-Based Battery Waste

**DOI:** 10.1021/acsomega.6c00180

**Published:** 2026-07-09

**Authors:** Jere Vänskä, Tiia-Maria Porkola, Lassi Klemettinen, Jere Partinen, Mari Lundström

**Affiliations:** 174277 Aalto University, School of Chemical Engineering, Department of Chemical and Metallurgical Engineering, Aalto 00076, Finland

## Abstract

In recent years, lithium iron phosphate (LFP) battery chemistries, both with and without manganese doping, have become increasingly common in battery applications. As the use of this battery chemistry expands, the number of end-of-life batteries will increase, highlighting the need for efficient recycling methods and knowledge of black mass characteristics. Thus, a comprehensive characterization of industrial lithium iron phosphate-based black mass was conducted. In this study, Li (3 wt %), Fe (12 wt %), P (6 wt %), and Mn (8 wt %) were detected in the black mass. Significant amounts (>1 wt %) of Co and Ni were also found, along with several doping agents (1600 ppm Ti, 1500 ppm V, and 690 ppm Si) and impurities (1400 ppm S). The size fraction with the highest mass after sieving (45 wt %) was 125–224 μm. However, characterization results showed that the particles were mainly agglomerated, and the actual dominant particle size is smaller than 125–224 μm. In smaller sieving size fractions (<125 μm), the carbon had a more graphitic structure, whereas in larger size fractions, the carbon had a lower graphitization degree and more defects, suggesting the presence of polymers. The results suggest high heterogeneity in the industrially provided black mass, highlighting the importance of developing a flexible process for future LFP-based battery recycling facilities.

## Graphical abstract

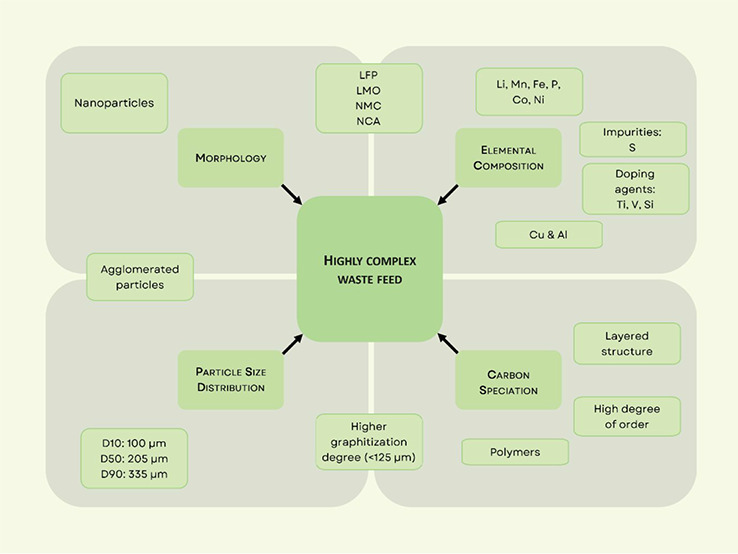

## 1 Introduction

Lithium iron phosphate (LFP) batteries have gained a solid position in the global battery market, especially within electric vehicles (EVs) and stationary energy storage systems (ESSs).^1^ LFP chemistry is utilized mainly due to its feasibility, safety, and long-term lifespan.^1^ Currently, high-nickel battery chemistries have the highest market share within the EV sector in Europe, but the share of LFP batteries has been increasing annually and shows no signs of reaching a plateau.^2^ Even though LFP-based batteries have gained popularity, they still lag behind other battery chemistries in some features, such as low conductivity and lower ionic diffusion compared to lithium nickel–manganese-cobalt oxide (NMC) batteries with high nickel contents, such as NMC811.^3^ To enhance the above-mentioned properties of LFP batteries, the cathode active materials are typically doped and/or coated with additives. The most common method to increase the electrical conductivity is to coat the LFP particles with an amorphous carbon layer a few nanometers thick.^4^ Doping of LFP with various metals has also been studied (Table 1). According to the available literature, the most common doping agents for the cathode are manganese, nickel, and cobalt. Additionally, the anode can be doped with silicon and titanium, for example.^5,6^ The combination of LFP and NMC cathode chemistries has also been studied.^7,8^

**Table 1:** List of Some Investigated Doping Agents and Their Effects on LFP and LMFP Batteries

Material	Amount (at.%)	Site	Effect	References
Al	1	Li	Improves conductivity, initial capacity and rate and cycle performance	^9^
Na	5	Li	Increases the conductivity, the rate and cycle performance, and Li^+^ deintercalation	^10^
Nb	2	Li	Promotes lithium diffusion and increases electronic conductivity	^11^
Ti	3	Li	Increased cycle performance and conductivity	^12^
Na and K	2 and 1	Li	Improves the reversibility of the electrode reaction and increases conductivity	^13^
Zr and Co	0.25 and 1	Li and Fe	Improves conductivity and cycle stability	^14^
Co	1	Fe	Increases capacity and Li diffusion	^15^
Cu	6	Fe	Reduces charge transfer resistance	^16^
Mg	0.43	Fe	Increases Li diffusion rate, improves conductivity	^17^
Mn	15	Fe	Increases conductivity	^18^
Mg and Ti	0.5 and 1	Fe	Improves diffusion coefficient and high-rate performance	^19^
V and Ti	7 and 3	Fe	Increases charge–discharge capacity	^20^
Ni, Co, and Mn	2.94, 0.3, and 0.3	Fe	Improves reversibility and cycling	^21^
V	3	Fe	Improves conductivity, cycling rate and stability	^22^
Cr	3	Fe	Improves rate performance	^23^
Zn	5	Fe	Shortens the ion transport path, increases Li storage capacity	^24^
Ni and Mn	2 and 3	Fe	Improves cycle and rate performance, improves Li diffusion and reduces charge transfer resistance	^25^
Y	1	Fe	Improves Li diffusion, stabilizing structure, decreases electrode resistance	^26^
Mo	5	Fe	Improves Li diffusion	^27^
B	2	P	Improves electronic conductivity	^28^

One promising method to improve the electrochemical properties of LFP batteries is to replace some of the iron with manganese to achieve the composition LiMn_*y*_Fe_1–*y*_PO_4_. It is estimated that lithium manganese iron phosphate (LMFP) batteries would gain popularity in EV markets due to their enhanced properties, e.g., higher energy density than conventional LFP batteries while still preserving structural and thermal stability compared to other lithium-ion battery chemistries.^1,29^ However, this comes at the cost of a reduced cycle life compared to conventional LFP batteries due to Mn dissolution into the electrolyte.^30^ According to the literature, there have been studies using an Mn content of *y* = 0.1–0.8,^31–33^ where it has been found that the optimum amount of Mn in LiMn_*y*_Fe_1–*y*_PO_4_ would be ca.*y* = 0.5–0.8.^8,32^ The above-mentioned effect of Mn in increasing energy density is a common reason for its use as a doping element in LFP, in conjunction with carbon coating to increase electrical conductivity.^32^

Another promising dopant is Zn, which increases the specific capacity, lithium diffusion rate, and also hinders Fe and Mn dissolution.^34^ Additionally, Mg, Nb, Ni, Co, Ca, Ti, V, and Cr have been investigated as potential doping agents on the Fe/Mn site mainly to increase the Li diffusion rate, conductivity, or to improve structural support.^35^ However, the very same doping metals make the recycling of batteries based on LFP and LMFP chemistries more complex by introducing more metals into the system at low concentrations. In state-of-the-art battery recycling processes, conventionally only a few doping metals are recovered, namely Co, Cu, Ni, and Mn.

In the framework of lithium-ion batteries, LFP and LMFP batteries have reasonably similar chemistries and share common physical, chemical, and electrochemical properties. Because of their similarities, it can be expected that LMFP batteries will be recycled on an industrial scale using similar processes as LFP batteries. Once a spent battery reaches the end-of-life stage, based on legislation and principles of circularity, it is required to be recycled.^36^ However, iron and phosphorus are excluded from the methodology to calculate recycling efficiency,^36^ and this has raised public discussion in Europe. For example, the European Commission received a letter from European battery recyclers and environmental nongovernmental organizations (NGOs) to highlight this loophole within the Battery Regulation (2023/1542).^37^ In the letter, it is noted that, for example, carbon, iron, and phosphorus may not be included in the 65% recycling target set for lithium-based batteries, which contradicts the environmentally ambitious EU targets within the Battery Regulation (2023/1542).^37^

In general, battery recycling is divided into two recycling routes: solely hydrometallurgical recycling and a combination of pyrometallurgical and hydrometallurgical recycling.^29^ The recycling routes containing pyrometallurgical processes benefit from the feature that battery waste does not necessarily require any pretreatment. The hydrometallurgical recycling routes can only treat pretreated (discharged, crushed, and sieved) battery waste, referred to as *black mass* (BM). However, according to Rinne et al.,^38^ the set recycling targets are currently not being achieved for all relevant elements with state-of-the-art pyrometallurgical processes. In addition to metallurgical recycling, so-called “direct recycling” has been suggested as a potential recycling method, although the technology is not mature enough to be used for the recycling of end-of-life battery waste.^39^ In hydrometallurgical LFP recycling, the economic feasibility is considered to be one of the greatest challenges,^40^ although technically, the set recycling targets of 65 wt % are potentially reachable using hydrometallurgical methods.

To gain more insights into the recycling of these phosphate-based batteries and specifically the black mass obtained after mechanical treatment of end-of-life battery cells, it is unequivocally crucial to understand the characteristics of this complex raw material itself. Therefore, a detailed characterization of black mass is a necessity. Although new applied analytical methods have been developed, there is only a limited amount of research related to the characterization of industrial LFP black mass and spent LCO batteries.^41–45^ Further, in many scientific publications, only certain metals—namely lithium and iron—have been analyzed from phosphate-based black mass. However, the black mass includes a variety of other elements, such as doping and coating agents, and therefore an extensive characterization is needed to design a recycling process that can truly support circulation of all relevant materials. Based on the literature survey, it is apparent that the thorough characterization of industrial LFP battery waste has not been widely published. The current study extensively investigates the composition, mineralogy, and structure of industrial phosphate-based black mass with the aim of providing knowledge that can help in building future strategies for recycling black masses obtained from phosphate-based battery chemistries. Detailed experimental research regarding possible recycling processes and process parameters will be conducted in later publications and is outside the scope of the current work.

## 2 Materials and Methods

The phosphate-based black mass sample was received from an industrial battery recycler, Stena Recycling. The material was obtained by discharging the end-of-life battery packs via an external circuit, followed by manual disassembly to battery module level. After this, the discharged modules were shredded in an inert atmosphere, dried to recover most of the electrolyte, and sieved via a vibratory table as described in open literature^46^ and stated by the company. The black mass was sieved using a vibratory sieve shaker (Fritsch, Analysette 3, Idar-Oberstein, Germany) with an amplitude of 5 mm for 30 min through varying sieve mesh sizes: 125 μm, 224 μm, 250 μm, 355 μm, 500 μm, and 1000 μm. The different fractions were weighed to obtain the particle size distribution (PSD). Additionally, the <500 μm fraction was also sieved for further analysis to gain a general overview of the black mass instead of fractions and to homogenize the studied sample.

To analyze the bulk elemental concentrations in different fractions, solution analysis was utilized—namely atomic absorption spectroscopy (AAS, Thermo Scientific iCE 3000, USA) and inductively coupled plasma–optical emission spectroscopy (ICP-OES, Agilent 5900 SVDV ICP-OES, USA). To dissolve the black mass fractions, they were leached with aqua regia (1:3 molar ratio of HNO_3_:HCl) at elevated temperature (70 °C) with stirring (300 rpm) on a heater with magnetic stirrer (IKA RT10, Germany) for 90 min. A total of 40 mL of aqua regia was used for 1 g of black mass. After aqua regia leaching, the samples were cooled down to room temperature and diluted to 50 mL with deionized water (15 MΩ·cm, Merck Elix Essential, France). Commercial chemicals were used alongside the industrial black mass. For total leaching, 65% HNO_3_ (Merck, Germany) and 37% HCl (VWR Chemicals, Belgium) were used. Additionally, 2% and 5% HNO_3_ solutions were used for sample dilutions. For semiquantitative elemental analysis of the black mass, inductively coupled plasma–sector field mass spectrometry (ICP-SFMS, Measurlabs, Helsinki) was utilized, which conformed to standard ISO/IEC 17025. Fluorine was analyzed by combustion ion chromatography (CIC) (Measurlabs, Helsinki), in compliance with method DIN 51723 Verf. B; 2006-06.

To investigate the structure and morphology of the black mass and the particles within it, a scanning electron microscope (SEM, Jeol JSM-IT800HL, Japan) was utilized, coupled with an energy-dispersive X-ray spectrometer (EDS, Oxford Instruments Aztec Live Premium Ultim Max 100, United Kingdom). Microstructural images were taken mostly with a secondary electron (SE) detector using a 5 kV acceleration voltage and a 9 pA beam current. The sample was prepared by placing a small amount of the LFP-based powder on double-sided carbon tape mounted on an aluminum sample stub. To investigate the elemental composition of the particles quantitatively with EDS, some of the powder was cast into epoxy resin, ground, polished to a 1 μm surface finish, and carbon coated (Jeol IB-29510VET, Japan). A 15 kV acceleration voltage with a 1.3 nA beam current was used for EDS point analyses. Elemental maps were obtained with 1024-pixel resolution, using a 400 μs analysis time per pixel in the Quantmap mode of the EDS software. Some elemental maps were also acquired with another SEM-EDS system (Tescan Mira3 SEM, Czech Republic, coupled to an Oxford Instruments AztecLiveLite with Xplore 30 EDS detector, United Kingdom). Before analysis, a beam measurement was performed using polished, pure Cu (grade A) as the calibration element. The beam measurement enables unnormalized, quantified results to be obtained using the factory standards database supplied by Oxford Instruments.^47^

The total carbon (TC) analyses were conducted using a Thermo Flash Smart CHNS elemental analyzer (Thermo Scientific, USA) with Eager Smart software. Each sample was weighed and analyzed five times, and BBOT (C_26_H_26_N_2_O_2_S) was used as a standard. The sample weight was 4–5 mg, and 8–10 mg of WO_3_ per sample was used as a catalyst. The carbon speciation was investigated using a confocal Raman microscope (Renishaw, inVia Qontor, UK) with a 532 nm wavelength and 5% laser power. The Raman shift was measured at 300–3000 cm^–1^. To estimate the carbon quality in the studied black mass fractions, the main peak ratios (*I*_D_/*I*_G_ and *I*_2D_/*I*_G_) were calculated based on the peak intensities.

The crystalline particles within the battery scrap were investigated via X-ray diffraction (XRD, PANalytical Aeris Benchtop XRD, equipped with a PIXel^1D^ detector, Cu–Kα radiation source, operated at 40 kV, 7.5 mA, along with a Kβ filter) and the obtained spectra were assessed and compared with software (HighScore Plus, Netherlands) utilizing the PDF-5+ database.

## 3 Results and Discussion

### 3.1 Particle Size Distribution (PSD) Based on Sieving

The PSD calculated after sieving showed that the D10, D50, and D90 of the black mass were 100, 205, and 335 μm, respectively (Figure S1). These results are larger compared to previous research related to NMC black mass characterization.^48^ However, the particles can agglomerate and therefore skew the results, and the particle shape may also somewhat skew the distribution. The cumulative PSD may indicate that the black mass had already been sieved during prior mechanical treatment based on the modest amounts in larger fractions, e.g., the >1000 μm fraction accounts for only 0.05 wt % of the total mass. But when comparing the PSD to previous LFP black mass characterization results, the distribution has similarities with the findings of Bjerre-Christensen et al.^45^ (Table S1). In both studies, the largest fraction by mass is in the particle size range of 125–250 μm.

### 3.2 Elemental Analysis of the <500 μm Fraction

In order to investigate the chemical composition of the black mass, the bulk sample (<500 μm black mass size fraction) was analyzed via ICP-OES, ICP-SFMS, AAS, CIC, and CHNS. The analysis indicates that the black mass contained Li (2.9 wt %), Fe (11.7 wt %), Mn (7.7 wt %), and P (6.2 wt %) (Table 2). Surprisingly, Ni and Co were also present in substantial quantities: 3.5 and 1.1 wt %, respectively. The existing literature reports mixing of NMC and LFP chemistries in some cases within the cathode active material (CAM).^7^ The concentrations of typical current collector materials Al and Cu were found to be low (1–1.5 wt %), which aligns with the compositions of sieved fractions reported in the literature.^41,45,49^ However, Al can also exist as a doping agent in cathode materials.^9^ The fluorine concentration in the <500 μm fraction was 2.1 wt %, indicating that traces of the electrolyte or binder remain in the black mass,^50^ whereas total carbon (TC) of 30.5 wt % matches previous research to some degree^49^ and it is estimated that most of the carbon originates from the anode coating. However, the carbon content can vary drastically based on the applied pretreatment methods, such as flotation.^51,52^

**Table 2:** Chemical Composition of the <500 μm Fraction Analysed Using Different Techniques (AAS, ICP-OES, ICP-SFMS, CIC, and CHNS)

	Li	Fe	P	Mn	Al	Cu	Ni	Co	F	C
**AAS**	2.6	11.3	-	7.7	-	1.6	3.8	1.2	-	-
**ICP-OES**	2.7	11.7	6.5	7.9	1.1	1.5	3.3	1.1	-	-
**ICP-SFMS**	3.4	12.0	5.8	7.4	1.0	1.3	3.4	1.1	-	-
**CIC**	-	-	-	-	-	-	-	-	2.1	-
**CHNS**	-	-	-	-	-	-	-	-	-	30.5
**Avg.**	2.9	11.7	6.2	7.7	1.0	1.5	3.5	1.1	2.1	30.5
**σ**	0.43	0.34	0.50	0.27	0.06	0.17	0.26	0.06	-	-

Elements at lower quantities were also detected in the <500 μm fraction by ICP-SFMS analysis (Table S2). For example, Ti (1600 ppm), V (1500 ppm), S (1400 ppm), Si (690 ppm), Zn (590 ppm), Ca (510 ppm), Na (290 ppm), Mg (200 ppm), Sn (180 ppm), B (120 ppm), Zr (120 ppm), Nb (96 ppm), Cr (54 ppm), K (44 ppm), Mo (3 ppm), and Y (2.2 ppm) were found in the black mass. As can be seen, the doping metals studied for LFP and LMFP batteries (Table 1), i.e., Ti, V, Zn, Ca, Na, and Mg, were found in moderate amounts (>100 ppm) in the black mass. Also, silicon is known to be a prominent material in LFP batteries on the anode side as a composite agent,^1,5^ and it was also found within the black mass in limited amounts. According to the literature, the detected Ti may have originated either from the cathode or from the anode, since Li_4_Ti_5_O_12_ (LTO) can be used as an anode coating material within LMFP batteries.^8^ In addition, sulfur can appear as an impurity in the precursor (pCAM) for batteries throughout the battery manufacturing process, since various metal sulfates are used in the pCAM manufacturing process—most commonly FeSO_4_ is used either in the solid-state synthesis or thermal liquid-state synthesis of LFP CAM.^53^

### 3.3 Elemental Analysis of Different Particle Size Fractions

To further investigate the deportment of different materials into specific size fractions, the sieved fractions (<125 μm, 125–224 μm, 224–250 μm, 250–355 μm, 355–500 μm, 500–1000 μm, and >1000 μm) were also separately analyzed by ICP-OES, AAS, and CIC (Figure 1). The elemental compositions were found to be relatively similar among each fraction: Li (1.0–2.1 wt %), Fe (4.1–10.1 wt %), P (3.1–7.0 wt %), Mn (3.0–7.1 wt %), Al (0.04–1.02 wt %), and Cu (0.05–1.04 wt %), and the total content of these elements in samples varied between 16 and 38 wt %, being lower in fractions >500 μm. The total amount of carbon was surprisingly constant (30.4–31.1 wt %) in all analyzed particle size fractions below 500 μm (Table S3). The total carbon analysis was not conducted at all for the size fractions >500 μm. The rest of each fraction (in addition to the analyzed metals and total carbon) is proposed to consist of oxygen and trace elements (Table S2), and in the fractions >500 μm, different carbon species were definitely also present, even though their amounts were not quantified. The existing literature^45^ suggested considerably greater compositional variations between different size fractions of LFP black mass. In their characterization, it was found that carbon was concentrated in the smaller size fractions, whereas Al and Cu were concentrated in the larger fractions.^45^

**Figure 1:**
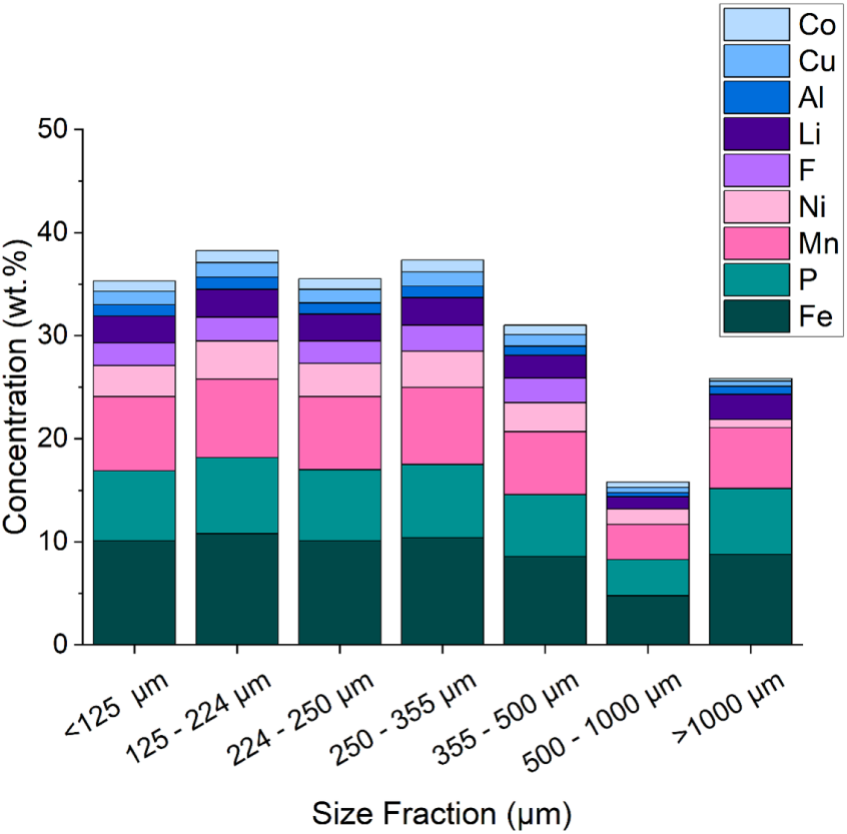
Elemental concentrations of Fe, P, Mn, Ni, F, Li, Al, Cu, and Co within different size fractions.

The similar compositional results for the different size fractions obtained in this work indicate that the sieving process did not effectively classify the individual particles (with varying compositions) into size fractions. Instead, most of the fractions contained the same particles, meaning that the sieving process mostly separated different-sized particle agglomerates into the respective fractions. This is further discussed in the next chapter in relation to the SEM-EDS analysis.

### 3.4 SEM-EDS Analysis of the <500 μm Fraction

The SEM-EDS characterization performed in this work was an iterative process. First, SE images were taken of the <500 μm powder sample attached to a double-sided carbon tape, in addition to preliminary qualitative EDS analyses on several particles to gain a better understanding of the variety of particle compositions found within the sample. Subsequently, powder of the same size fraction was cast into epoxy, ground, polished, and carbon-coated to create an even surface, enabling quantitative EDS results to be acquired. Additionally, several backscattered electron (BSE) images were obtained to highlight the compositional differences between particles (BSE contrast based on average atomic number). The quantitative analyses revealed several particles or particle agglomerates with interesting compositions. To match the compositions with the SE images taken of the powder without epoxy casting, more (qualitative) EDS data were obtained from the powder sample on carbon tape to improve the understanding of the size, shape, and general morphology of particles with different compositions.

SEM-SE images of the black mass (<500 μm) showed that the black mass contained particles with different shapes and sizes, e.g., spherical, flaky, or cylindrical. The SEM graphs (Figure 2) reveal that the vast majority of the particles were smaller than 20 μm and that there were clear agglomerates, which indicates that the PSD is in fact much smaller than the above-mentioned PSD results based on sieving. During sample preparation onto double-sided carbon tape, the larger agglomerates broke relatively easily when the aluminum sample stub was knocked against a table.

**Figure 2:**
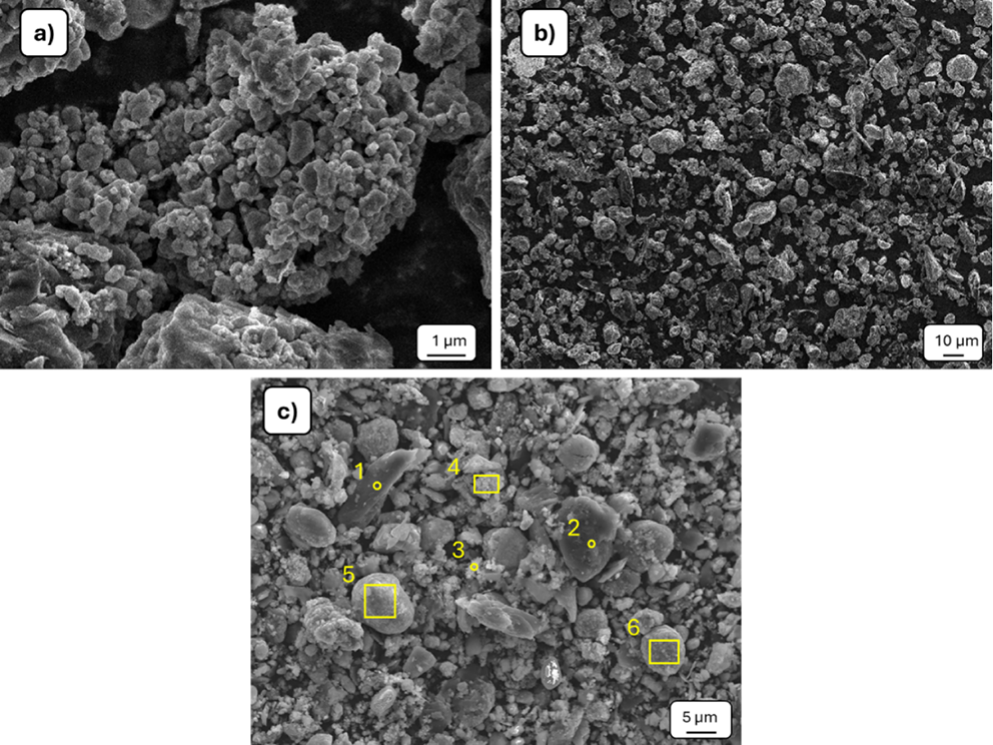
SEM micrographs of (a) agglomerated cathode material particle, (b) overview of <500 μm size fraction, and (c) another overview of <500 μm size fraction, with numbers indicating the areas where qualitative EDS analyses were performed.

The black mass (Figures 2 and S2) seems to contain both relatively spherical particles and flakier particles from the cathode side, possibly indicating that different synthesis methods are used on an industrial scale. The larger particles—Figure 2c, points 1 and 2—are carbon, based on qualitative EDS analysis performed on the uneven surface of the powder material. On the basis of the SE images (Figure 2c), the shape of these carbon-containing particles is mostly spherical, indicating particle surface modification to achieve better properties for the anode active material.^54^ The particles denoted by points 3 and 4 contain mainly Fe, P, and O (as well as C), indicating clearly that these are LFP particle agglomerates. Point 4 also contained trace amounts (<1 at. %) of possible doping elements Mn, Ti, and V. The particles numbered 5 and 6 were also spherical and included high concentrations of Ni, Co, and O, along with some Al and F. However, only trace concentrations of Mn (and low amounts of Fe and P) were found in these particles. Based on these results (high Ni, lower Co, and even lower Al), the particles numbered 5 and 6 were likely lithium nickel cobalt aluminum oxide (NCA). Some fibers were also observed in the black mass (Figure S3), although their compositional analysis was difficult due to instability under the electron beam.

Examples of quantitative EDS results from the sample cast in epoxy, polished, and carbon-coated are presented in Tables S4–S5. The areas where EDS data were taken are also shown in Figure 3. Area 1 (Figure 3a) contained higher concentrations of Ni, Mn, Co, and O with an atomic ratio of 1:1:1:5.7, which clearly indicates an NMC111 particle (Li_1_Ni_0.33_Mn_0.33_Co_0.33_O_2_). Additionally, the shape and size of the particle are similar to those of NMC particles reported in the literature.^7,55,56^ Areas 2–4 contained significant amounts of Mn and O with Mn:O atomic ratios of 1:1.9, 1:2.1, and 1:1.9, respectively. Ni and Co were not observed in these particles, which would indicate the presence of Mn_2_O_4_, or more likely LiMn_2_O_4_ (LMO) cathode material. The shapes of these particles are flakier and more irregular compared to the NMC111 particle; however, the exact shape is difficult to determine from the polished section. Therefore, some qualitative EDS analyses were performed on a powder sample without epoxy casting to identify similar particles and observe their morphologies (Figure S4). When comparing the sample to an SEM-SE image of LMO powder reported by Romero-Núñez and Ibarra-Palos,^57^ similar polyhedral shapes can be observed. The individual particle located toward the top (Figure S4a) has better-defined edges, whereas it seems that the (Li-)Mn oxide particles at the center of the figure are embedded in some sort of binder. The qualitative EDS data also supports this statement, as the F concentration in that area was high (>20 at. %). An LMO particle agglomerate is also seen, in which the shapes of individual particles are clearer due to the presence of less binder.

**Figure 3:**
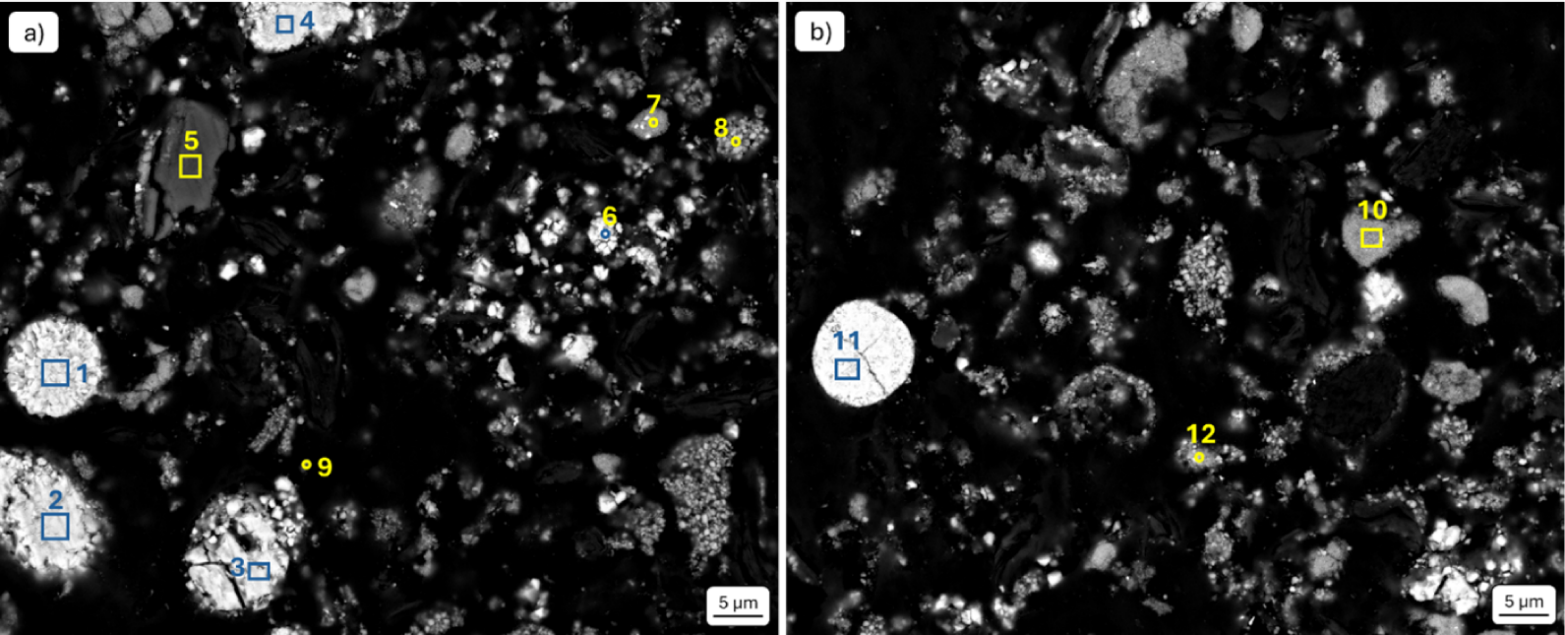
SEM-BSE micrographs (a) and (b) of the particles from the <500 μm fraction cast into an epoxy button. Quantitative EDS results of the areas marked with numbers 1–12 are presented in Supplementary Tables S4–S5.

Excluding trace elements below 0.5 wt %, the larger particle denoted by area 5 (Figure 3) was composed of only Al and O, which indicates that the Al from the current collector had been oxidized. The small particle cluster at point 6 contained mainly Ni and Co oxides with some Al, indicating again particles with NCA chemistry. Points 7 and 8 contained significant amounts of Fe, P, and O, with Fe:P:O ratios of 1.1:1:4.4 and 1:1:3.4, respectively. Based on this, these small agglomerates are composed of LFP. The small particle size also emphasizes that the material at points 7 and 8 is LFP, as the size of individual particles typically ranges between 0.1 and 2 μm.^58,59^ The sizes of LFP particles observed in this work (Figures 3a and S2) fall clearly within this range, with typical diameters between 0.2 and 1 μm. Analysis point 8 contained a significant amount of Si (∼7 at. %), which is typically used as a doping agent on the anode side in LIBs.^5^ The analysis results of this particular point are heavily affected by the small size of the particle, as evidenced by the high carbon concentration, which mostly originates from the coating as well as the epoxy. During qualitative EDS scanning of different particles in the LFP-based powder without epoxy casting, a few additional particles with high Si concentrations were found (Figure S5). Some of these particles had irregular shapes (Figure S5a), although some were shaped more like rods or cylinders (Figure S5b).

From another analysis location on the polished cross-section sample (Figure 3b), a highly spherical particle with a high atomic number (area 11) was found. This particle fits well with NCA cathode chemistry (LiNi_0.8_Co_0.15_Al_0.05_O_2_), as it contained elevated amounts of Ni (∼28 at. %), lower amounts of Co (∼5 at. %), as well as small amounts of Al (∼2 at. %) with only trace amounts of Mn (<0.5 at. %). A higher concentration of F (∼7 at. %) was also observed, which could indicate either traces of PVDF from the binder or LiPF_6_ from the electrolyte.^60,61^

Most of the analyzed LFP particles in the sample did not contain Mn above trace quantities. However, at point 12, elevated amounts of both Fe (1.2 at. %) and Mn (∼10 at. %) were detected. The Fe:P atomic ratio was 1:1.5, whereas the Mn:P ratio was 5.6:1. When performing the qualitative EDS analyses of powder material, some other particles or agglomerates with Fe, P, and Mn (as well as O) were also found. Usually, these particles also contained significantly higher concentrations of Mn compared to Fe and P, even though these analysis results are not quantitative due to the uneven surface of the particles. However, some particles were also detected where the Fe:P atomic ratio was roughly 1:1, with lower Mn concentrations (Mn = 0.15–0.4) (Figure S6).

As shown in Table 1, Ti has also been investigated as a doping element for LFP batteries. In the EDS analyses of this work, in general, Ti was not detected, even in trace amounts. However, high concentrations of Ti (oxides) were observed in one instance in small particles close to an LFP agglomerate (Figure S8), in addition to one larger (∼15 μm) agglomerate of Ti oxide particles (with high F concentration).

To visually illustrate the distribution of elements between different particles, quantitative EDS mapping was performed for the polished cross-section sample. As mentioned previously and further evidenced by the mapping results (Figures 4, S7 and S8) as well as quantitative point analyses (Tables S4–S7), surprisingly, Mn and Fe are mostly not located in the same particles. Looking at the distribution of Mn, Fe, O, and P, the majority of the Mn seems to be present as MnO_2_ or Mn_2_O_4_ (while Li cannot be analyzed using EDS), which strongly suggests that a significant quantity of LMO cathode material particles is present in the phosphate-based black mass. This may be due to the LMO chemistry being mixed with LFP within the same batteries, or issues during the mechanical sorting stage before crushing. Another surprising fact is that the majority of the Ni- and Co-containing particles did not contain any notable amounts of Mn (Figure 4 and Tables S4 and S7); however, in some particles Mn was located with Ni and Co (Figure 3, Tables S4 and S7), which supports the previous statement about NMC particles being present in the black mass. Most of the Ni- and Co-containing particles also had low concentrations of Al (2–3 at. %), indicating that NCA particles were also mixed in the phosphate-based black mass. As clearly seen from the elemental EDS maps (Figures 4, S7, S8), the majority of the particles contain Fe, P, and O. Therefore, even though numerous particles with various Li-ion battery cathode chemistries were evidently present, the black mass was heavily phosphate-based.

**Figure 4:**
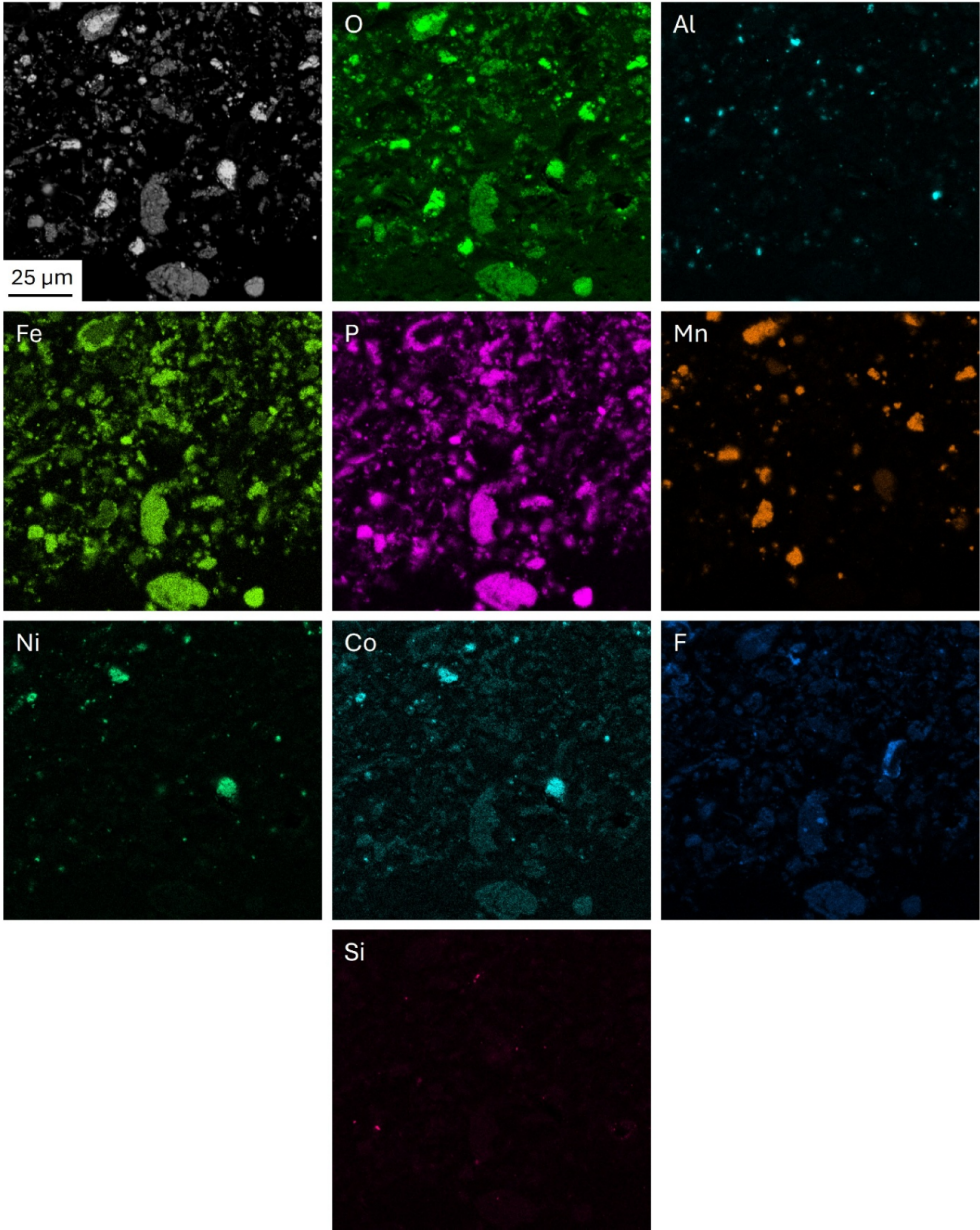
Elemental EDS maps of the <500 μm size fraction from a polished section. The maps clearly show that Fe and P are present in the same particles, and Mn was generally found in different ones. The Ni-rich particles also contained Co, sometimes with Mn, but mostly with low concentrations of Al.

If the other Li-ion battery chemistries were mixed at the cathode, the phosphate-based black mass would contain particles or particle agglomerates with more than one chemistry. An example of a case where two chemistries are present in the same agglomerate is shown in Figure S9 and Table S6, where NCA is present with LFP. Another interesting area can be seen at the top of Figure 4, where Fe, P, and Mn seem to be present in the same particle, but Mn concentration is higher at the center, whereas Fe and P are located more toward the edges. A close-up of this particle is shown in Figure S10, and the EDS analysis results are given in Table S7.

### 3.5 Raman Spectroscopy of Different Size Fractions

The <125 μm, 355–500 μm, and <500 μm size fractions were further subjected to Raman spectroscopy to gain insights into the structure of carbon in the different size fractions. The main focus of the Raman spectroscopy was on the intensities of carbon-specific peaks (D, G, and 2D) (Figure S11). Especially, a high and sharp 2D peak indicates that the carbon structure is multilayered. Typically, intensity ratios of these peaks are used to assess carbon quality. A low *I*_D_/*I*_G_ ratio indicates a low defect density in the structure, while a high *I*_2D_/*I*_G_ ratio indicates a more ordered structure, further indicating higher carbon purity and quality.^62–64^ The <500 μm and 355–500 μm fractions exhibit similar broad peaks at relatively low intensities, whereas the fraction of <125 μm has sharp and strong peaks, which gives the fraction the lowest *I*_D_/*I*_G_ ratio (0.359) but also the highest *I*_2D_/*I*_G_ ratio (0.827). This suggests that the carbon in the <125 μm fraction has a highly organized structure and contains greater amounts of graphitic carbon. This observation is consistent with literature findings that most of the graphite is distributed in smaller particle size fractions.^45^ In other fractions—<500 μm and 355–500 μm—the carbon was found to have more disorder and contained more amorphous carbon, as seen from the broader and overlapping D and G peaks. Therefore, the lower-quality carbon in the larger fractions could be partially caused by longer fibers (Figure S3), possibly originating from casing and separator. However, the Raman spectroscopy indicates that graphite-like structure could be the most dominant carbon compound, also in these larger fractions. This may indicate that graphite adheres to Cu and Al particles and therefore ends up in larger size fractions also.

### 3.6 XRD of the <500 μm Size Fraction

The XRD analysis results obtained showed a few clear peaks indicating the crystal structure of some compounds found in the black mass (Figure 5). At the 2θ angle of ca. 26°, there was a significant peak, possibly comprised of multiple overlapping peaks. The possible peaks were deconvoluted and, according to the calculations, LMO, carbon, LFP, SiO_2_, LMFP, and LMP were found. Based on these analyses, as well as the SEM-EDS results (Figures 3, 4 and S4), it can be concluded that the presence of LMO is evident. The wide peak at ca. 26° could also indicate the presence of nanoparticles.^65^ This also reinforces the observation (Figures 2a, S2, S6) of LFP nanoparticles.^58,59^

**Figure 5:**
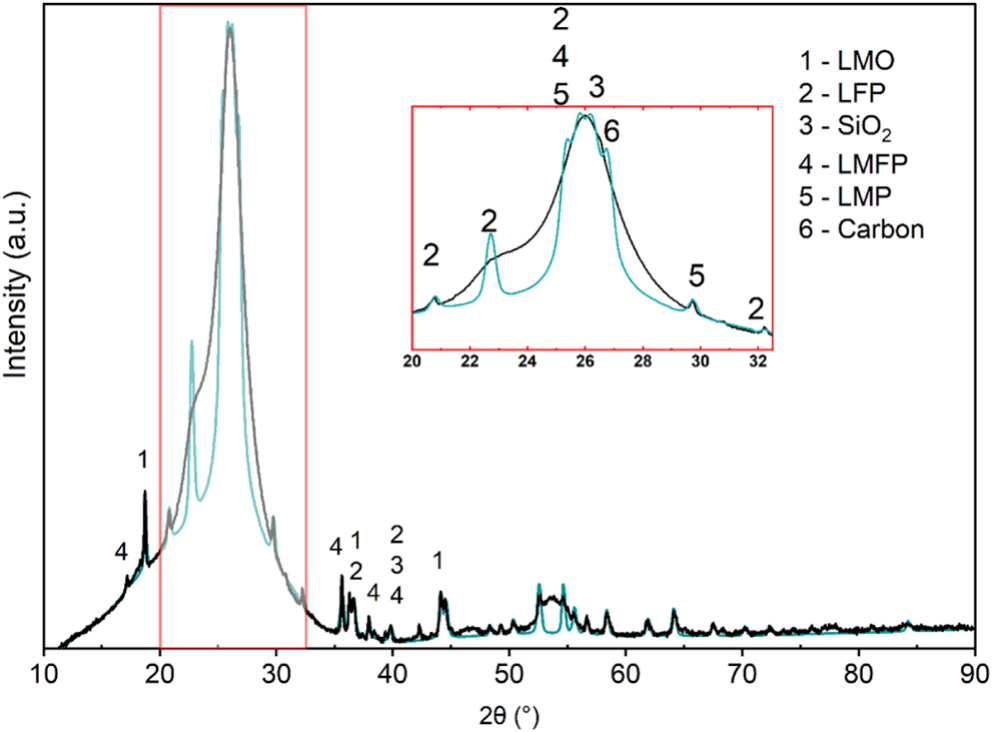
XRD graph of the <500 μm fraction. The black line represents the measured intensity, and the blue line expresses the calculated intensities. The *y*-axis scale is logarithmic to highlight the peaks.

LFP and LMFP share a similar XRD peak profile with only small variations.^66^ Based on these small variations, it can be seen that peaks in the 2θ range of 54–57° and 60–63° (highlighted with gray boxes in Figure 6) correspond to the characteristic X-ray peaks of LFP and two different LMFP compositions. For example, the peak at 54.6° is most likely attributed to LMFP with high Mn content, whereas the peak at 55.5° fits best LFP. However, as clearly seen from the reference peak locations illustrated in Figure 6, the peak positions of LFP and LMFP are extremely close to each other, making detailed and reliable characterization using XRD very difficult.

**Figure 6:**
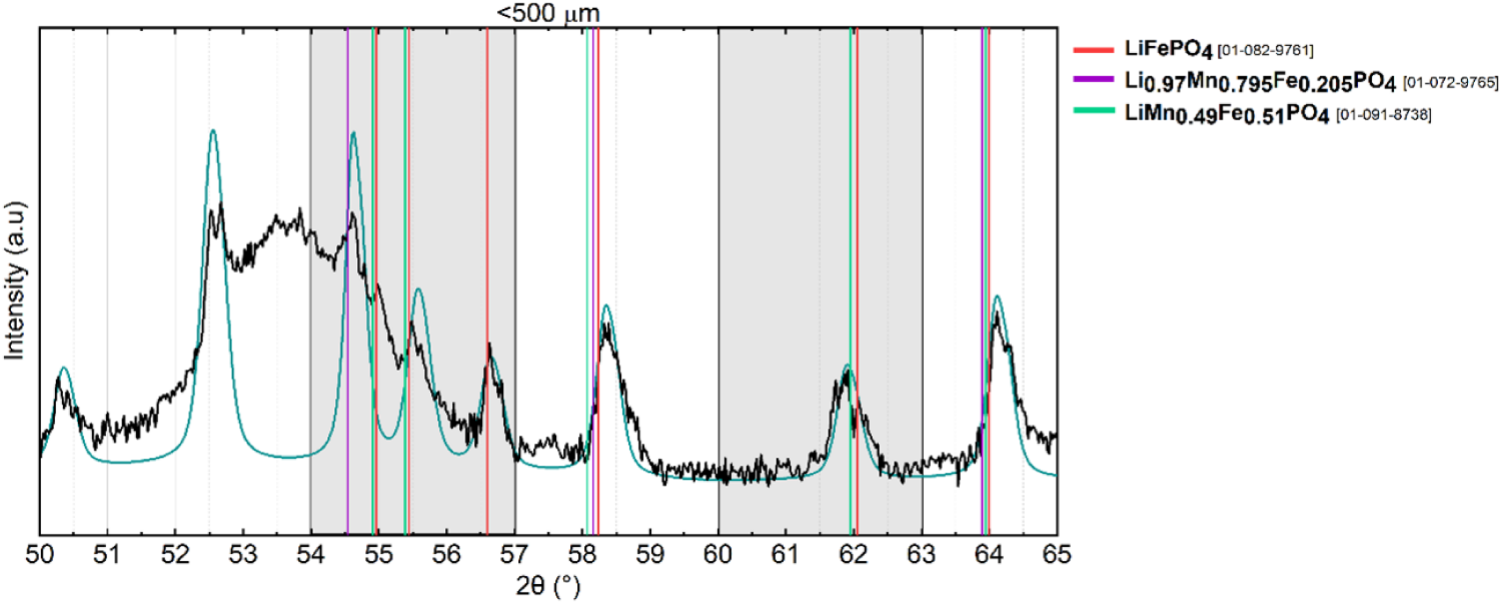
Highlighted XRD peaks of the <500 μm size fraction at the 50–65° 2θ range. Black lines represent the measured intensity, blue lines express the calculated intensities, and the vertical lines (red, purple, and green) illustrate the reference peaks (PDF card numbers are given in square brackets).

Despite the challenges in XRD characterization, the findings strengthen the statement that the industrial black mass comprises not only a certain phosphate-based battery chemistry but rather a mixture of different phosphate-based battery chemistries, as well as minor quantities of other Li-ion battery chemistries (Figure 5).

### 3.7 Industrial LFP-Based Black Mass Mineralogy and Chemistry

Table 3 summarizes the analyzed elements present in the raw material as well as their potential origin and mineralogy in the investigated black mass. The results suggest that significant amounts (wt %) of the analyzed elements (Li, Fe, P, Mn, Cu, Al, C, Co, and Ni) are used in the electrodes, either as a CAM or current collector. From these, some may also exist in the casing materials (Fe and Ni)^67^ and in the electrolyte (Li and P).

**Table 3:** Analyzed Element Concentrations, Potential Origin, and Mineralogy in the Industrial Black Mass^a^

	Amount	Origin	Main mineralogy	Other mineralogies
**Fe**	12 wt %	Cathode – main element	LFP	LMFP, Alloy
**P**	6 wt %	Cathode – main element	LFP	LMFP, LiPF_6_
**Li**	3 wt %	Cathode – main element	LFP	LMFP, LiPF_6_
**Mn**	8 wt %	Cathode – main element or doping agent	LMO	LMFP
**Ni**	4 wt %	Cathode, accumulation, casing	NCA	NMC
**Co**	1 wt %	Cathode or accumulation	NCA	NMC
**Al**	1 wt %	Electrode, cathode, doping	NCA, metallic	Ion
**Cu**	2 wt %	Electrode	Metallic	-
**F**	2 wt %	Electrolyte, binder, doping	PVDF	LiPF_6_
**C**	31 wt %	Anode, cathode coating, others	Graphite	Amorphous, polymers
**Ti**	1600 ppm	Anode or doping	LTO	Ion
**V**	1500 ppm	Doping	Ion	-
**S**	1400 ppm	Impurity	-	-
**Si**	690 ppm	Anode	SiO_2_	-
**Zn**	590 ppm	Doping	Ion	-
**Ca**	510 ppm	Doping	Ion	-
**Na**	290 ppm	Doping	Ion	-
**Mg**	200 ppm	Doping	Ion	-
**Sn**	180 ppm	Solder	Metallic	
**B**	120 ppm	Electrolyte or doping	LiBF_4_, LiBOB	Ion
**Zr**	120 ppm	Doping	Ion	-
**Nb**	96 ppm	Doping	Ion	-
**Cr**	54 ppm	Doping, casing	Steel alloy	Ion
**K**	44 ppm	Doping	Ion	-
**Mo**	3 ppm	Doping	Ion	-
**Y**	2.2 ppm	Doping	Ion	-

a Doping metals (“Ion”) replace either Li, Fe , or P in the orthorhombic structure.

The most dominant cathode chemistry in the black mass was that of LFP. Interestingly, Mn was found in multiple different phases, mostly as an oxide but also as LMFP, indicating that it exists in the battery waste integrated into the iron phosphate structure but mainly as separate (lithium) manganese oxide particles, most likely originating from LMO cathode chemistry or, to a smaller extent, from different NMC chemistries. Carbon was found to have graphitic, amorphous, and polymeric structures. Graphite originates mainly from the anode, whereas amorphous carbon may originate from the LFP coating^4^ and polymeric structures may originate from the binder, separator, or casing.^68^ In addition to carbon, on the basis of the literature and the results of this research, anode materials may contain Si, mostly as an oxide.

Most of the elements present in minor quantities are likely to be doping elements of LFP or LMFP, and these are typically located on the Fe site within the LFP structure (Ti, V, Zn, Mg, Y). However, some of these can also be located on the Li site (Ca, Na, Zr, Nb, K) or even on the P site (B) in LFP chemistries (Table 1). One exception is chromium, which is suspected to originate from the steel casing; however, potential use as a dopant is not fully excluded. From the above-mentioned doping elements, only vanadium (Tables S4–S7), zirconium (Tables S6–S7), tiny amounts of calcium (Tables S6–S7), and titanium (Figure S6) were observed via methods other than ICP-SFMS.

The traces of electrolyte still present in the black mass were mainly identified based on fluorine. Fluorine traces (2.1 wt % total concentration in <500 μm fraction, Table 2) were found to cover some of the particles based on the SEM-EDS results (Tables S4–S7). Additionally, the aforementioned boron was suspected to also be used in the electrolyte,^69^ although it is a known doping/coating agent also in NMC chemistries.^70,71^ Sulfur was mainly considered as an impurity, possibly originating from the manufacturing of the pCAM, and was found in trace amounts (1400 ppm) in the black mass.^53^

## 4 Discussion

The development of feasible recycling routes for LFP-based battery waste is a challenge due to economic constraints related to the materials used and their value. Regarding the state-of-the-art pyro- and hydrometallurgical recycling processes, Cu, Ni, and Co are well recovered. Li and Mn recovery have been addressed during the last few years, but iron and phosphorus recovery is still largely overlooked, resulting in the loss of these elements from circulation.^38^ Especially in the case of LMFP batteries or a mixed feed of LFP- and LMO-based chemistries, Mn recovery is even more crucial to acknowledge. The unexpected finding of the current work suggests a clear presence of Ni and Co in the LFP battery waste, mostly as NCA; however, the recovery of these elements alone, alongside Cu and potentially graphite, cannot yet raise the recovery rates up to EU Battery Regulation targets.^36^

The significant presence of doping agents was also evident in the LFP-based battery wastes investigated. Although present at low concentrations, they can potentially contaminate the high-purity products of battery recycling processes. Therefore, along with changes in battery chemistries, increasing focus needs to be placed on their characterization and separation. Nevertheless, if recovered, the feasibility of LFP recycling processes could possibly be enhanced and/or access to some of critical raw materials could be strengthened. For example, if titanium and vanadium could be recovered, they could potentially be used in current and emerging battery chemistries, such as sodium-ion batteries (SIBs), NMC battery coatings, or redox flow batteries.^1,72,73^

As hypothesized, fluorine was also present in the investigated black mass and was found at very high concentrations in some particle agglomerates, which could be explained by the presence of PVDF binders. During hydrometallurgical processes, fluorine could form complexes with metals, especially with aluminum—e.g., AlF_3_—which may cause unwanted coprecipitation even at a low pH.^74^ Alternatively, fluorine may hinder hydroxide or phosphate precipitation of aluminum due to the formation of stable complexes even at a relatively high pH of 4.5.^75^ On the other hand, in direct contact with water, LiPF_6_ can form aqueous or gaseous hydrofluoric acid, posing a severe safety issue.^76^ Based on simulation, hydrofluoric acid can also be formed in neutral or acidic conditions in the absence of Al^3+^.^77^ Also, in pyrometallurgical processes, fluorine is an issue, as it ends up in off-gases^78^ and therefore necessitates proper gas scrubbing during off-gas treatment. It is clear that the fluorine issue needs to be addressed in any recycling process developed—both for NMC and LFP chemistries—either during hydro- or pyrometallurgical treatment or already mitigated via pretreatment prior to metallurgical processing.^79^

Alongside battery metals, the recycling of both graphitic and amorphous carbon is often an overlooked topic within battery recycling, even though it is a critical raw material.^36^ Based on the insights of the current research, the fine-quality carbon—highly structured and graphitic—is mostly located in the smaller size fractions (<125 μm), whereas the more amorphous and poorer-quality carbon is located in the larger fractions. Therefore, the recovery of high-grade graphitic carbon from the fine fractions can be recommended, e.g., via froth flotation. However, the particle size of the studied black mass may even be too small (after deagglomeration) for efficient flotation processes. Additionally, carbon coating of LFP and LMFP particles may alter their surface properties, posing challenges for graphite recovery via flotation. Alternatively, graphitic carbon present in the leach residues could be valorized into other carbon compounds, such as graphene oxide.^80,81^

The results of the current study suggest that in the near future, the recycling industry may face a more complicated and heterogeneous phosphate-based black mass raw material than has been expected. This will demand the development of increasingly flexible recycling processes as well as new unit processes, enabling the recovery of low elemental concentrations of target elements from multimetal heterogeneous raw materials. Reaching the EU battery regulation targets will be challenging not only for pyrometallurgical^38^ but also for hydrometallurgical LFP recycling processes. This emphasizes the need for iron and phosphorus recovery as well as graphite valorization. Nevertheless, in light of the results of this study, more holistic recycling strategies are needed to address the complexity of phosphate-based black-mass processing.

When reflecting on the obtained characterization results from a recycling process design perspective, the leaching step should be both chemically and economically efficient. Additionally, the recovered materials should be valorized. One plausible approach to recycling LFP-based black mass is to use a low concentration of sulfuric acid. When using low sulfuric acid concentrations, Li and Fe are leached, but it is possible to mitigate the dissolution of Cu and Al^82^ and thus decrease chemical consumption. To recover the residual Fe and Al, a selective precipitation method can be utilized.^83^ In addition, Mn and the high-value metals such as Ni, Co, and Cu, can be recovered with various recovery methods such as solvent extraction, precipitation, and electrowinning.^84,85^ The leaching residue, mainly consisting of graphite, can be valorized to be used as a catalyst.^80,81^ However, the recycling process of the investigated LFP-based black mass must be studied as a whole. Experiments regarding the effects of leaching parameters and additives, as well as the effect of pretreatments such as pyrolysis, on the recovery of elements from the industrial LFP-based black mass are currently ongoing.

## 5 Conclusions

In this work, industrial LFP-based black mass was extensively characterized. According to the results, Li, Fe, P, Mn, Ni, Co, Cu, Al, F, and C were the main elements present in the battery waste (1–31 wt % each) with a varying particle size (from hundreds of nanometers up to a few hundred micrometers). The agglomeration of nanosized particles also caused some skewness in the particle size distribution obtained via sieving. The presence of Mn in such high concentrations was not expected, and Mn was found to be present in significant quantities in particles with LMO, but also LMFP and NMC chemistries. The presence of Co and Ni (>1 wt %) as oxides was initially surprising; however, it became evident that particles with NCA cathode chemistry were also present in the black mass. This reflects well the nature of the challenges the recycling industry is currently facing: the streams entering the recycling processes are very heterogeneous, potentially due to challenges in waste sorting or using the same pretreatment lines for various batteries.

The particle size of the cathode material in the investigated black mass was found to be fine (a few micrometers), and the larger particles often seemed to consist of agglomerated fine particles. The black mass was found to contain a broad range of different elements, many of which were identified as minor elements originating from the doping or coating agents used in the cathode or anode materials. The carbon content was evenly distributed (ca. 31 wt %) between the fractions below 500 μm in size; however, the more structured graphitic carbon was mostly located in the smaller size fraction (<125 μm), whereas the less structured carbon—potentially originating from the casing and separator materials—tended to be distributed into the coarser fractions.

The current research shows that elemental analysis alone does not provide sufficient information to allow for the design of industrial recycling processes, as several of the battery elements can be present in different species, requiring different leaching approaches. For example, Mn can be present in cathode oxide materials requiring a reductant or in olivine-structured LMFP structures, which are fully acid-dissolvable. Additionally, metallic Al originating from current collector foils requires an oxidant; however, Al from NCA chemistry and Al-based doping or coating materials may exist in acid-dissolvable forms (e.g., Al_2_O_3_). Fe also exists in fully acid-dissolvable forms (LFP and LMFP) but may need an oxidant if it originates from casings (metallic Fe).

The results of the current study clearly highlight the heterogeneous nature of industrial LFP-based battery waste, which may pose challenges for the EU battery regulation recycling targets if operated at state-of-the-art recycling plants. Therefore, intensive development of new robust, flexible, and economically feasible recycling technologies is needed to mitigate the low-value and complex nature of this battery waste type. It is crucial to focus on the feasible recovery of the major elements (Li, Fe, P, Mn, and graphite) but also to increasingly focus on the development of recovery of minor elements and prepare for the fact that current and future industrial LFP battery waste is a mixture of different cathode chemistries.

## Supplementary Material



## Notes

Particle size distribution graph and table (Figure S1 and Table S1), ICP-SFMS and CHNS analysis results (Table S2), bulk elemental concentrations within each size fraction (Table S3), additional SEM graphs of black mass (Figures S2–S6), EDS elemental mapping of black mass (Figures S7 and S8), quantitative EDS results of several particles in the black mass (Figures S9, S10, Tables S4–S7), and Raman spectroscopy results for three size fractions (Figure S11) (PDF)
