# Supplementary material for: Characterization of Industrial Lithium Iron Phosphate-Based Battery Waste
Source: ACS Omega. 2026 Jul 9;11(28):41456–67. doi: 10.1021/acsomega.6c00180 (PMC13392884; doi:10.1021/acsomega.6c00180)
Supplement: Supplementary file 1 [file ao6c00180_si_001.pdf]

Supplementary material for:

Characterisation of industrial lithium iron phosphate-based battery waste

Jere Vänskä, Tiia-Maria Porkola, Lassi Klemettinen, Jere Partinen, Mari Lundström

Aalto University, School of Chemical Engineering, Department of Chemical and Metallurgical Engineering, 00076 Aalto, Finland

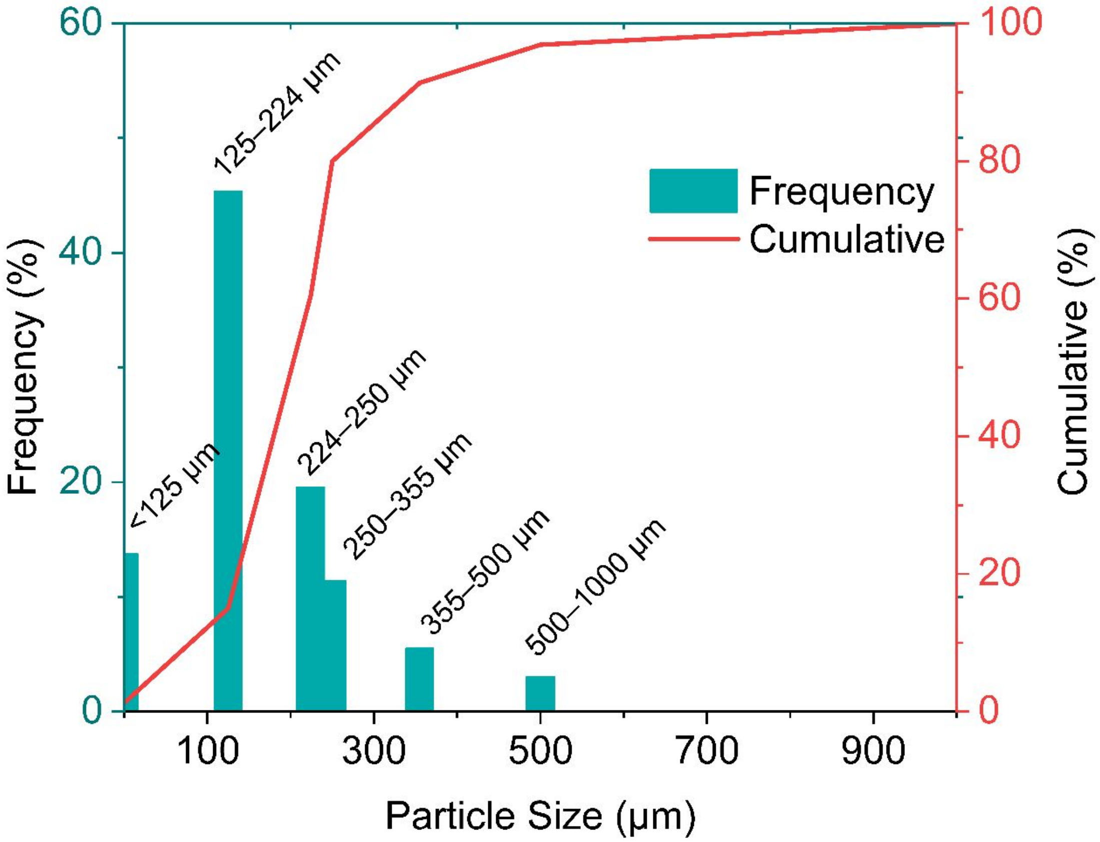

Figure S1. Cumulative and frequential PSD of the LMFP black mass. The columns correspond to the smaller particle size of each cut-off fraction.

Table S1. Frequential particle size distribution of the LMFP black mass (wt%). Results from Bjerre-Christensen et al. [45] are shown for comparison.

**<125 µm**	**125–250 µm**	**250–500 µm**	**500–1000 µm**
**This study**			
13.75	64.96	16.91	3.07
**Bjerre-Christensen *et al.*** [45]			
27.57	60.11	9.20	3.05

Table S2. Analysis results from ICP-SFMS for <500 µm particle size fraction, performed by an external partner. Additionally, H, N and S concentrations from CHNS analysis are presented.

Element	Concentration (mg/kg)	Element	Concentration (mg/kg)
Ag	5,3	Mo	3
Al	10000	N	1680
As	1,9	Na	290
Au	0,096	Nb	96
B	120	Nd	0,33
Ba	33	Ni	34000
Be	0,15	P	58000
Bi	1,9	Pb	36
Br	< 300	Pd	< 0.2
Ca	510	Pt	0,17
Cd	0,39	Rb	0,33
Ce	5,2	Rh	0,25
Co	11000	S	1400 (ICP-SFMS), 1290 (CHNS)
Cr	54	Sb	80
Cu	13000	Sc	0,15
Fe	120000	Se	< 0.5
Ga	3	Si	690
Ge	0,5	Sn	180
H	8730	Sr	5,1
Hf	2,6	Ta	0,12
Hg	< 0.1	Ti	1600
I	< 10	V	1500
K	44	W	30
La	0,84	Y	2,2
Li	34000	Zn	590
Mg	200	Zr	120
Mn	74000		

Table S3. Elemental concentrations within each analysed size fraction of the LMFP black mass.

	Li	Fe	P	Mn	Al	Cu	Ni	Co	F	C	Others*
**<125 µm**	2.6	10.1	6.8	7.2	1.1	1.3	3	1.01	2.2	31.1	33.60
**125–224 µm**	2.7	10.8	7.4	7.6	1.2	1.4	3.7	1.18	2.3	30.4	31.47
**224–250 µm**	2.6	10.1	6.9	7.1	1.1	1.3	3.2	1.03	2.2	30.7	33.76
**250–355 µm**	2.7	10.4	7.1	7.5	1.1	1.4	3.5	1.13	2.5	30.9	31.78
**355–500 µm**	2.2	8.6	6	6.1	0.9	1.1	2.8	0.9	2.4	30.9	38.05
**500–1000 µm**	1.2	4.8	3.5	3.4	0.4	0.5	1.5	0.5	-	-	84.30
**>1000 µm**	2.4	8.8	6.4	5.9	0.8	0.5	0.8	0.26	-	-	74.19

*includes carbon, oxygen and trace elements but in fractions <500 µm includes only oxygen and trace elements

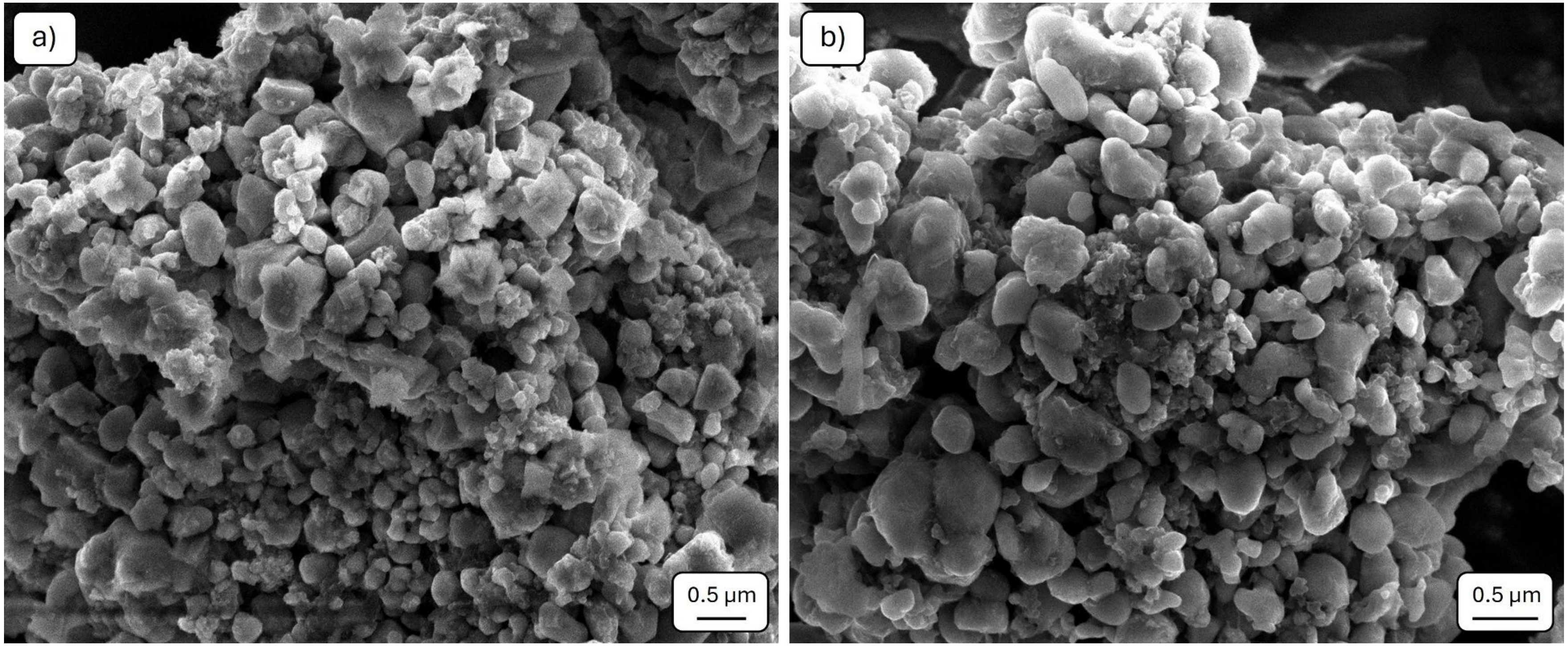

Figure S2. SEM-SE images of LFP-based cathode material particle agglomerates a) and b) observed in the <500 µm size fraction sample.

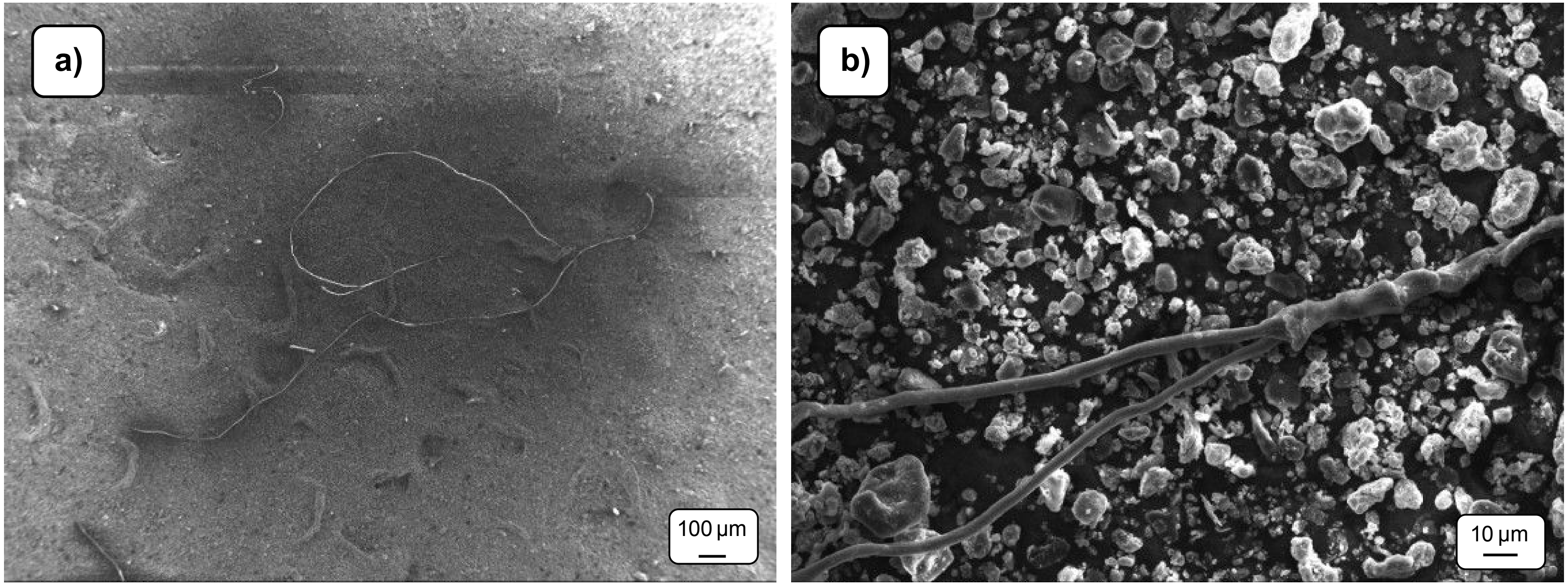

Figure S3. Observed fibres a) and b) in the <500 µm size fraction of LMFP black mass.

Table S4. Unnormalised wt.% and at.% results from the EDS analyses of points and areas indicated in Figure 3a. For analysis locations indicated with labels 1–6, carbon was left out because it was believed to originate from the carbon coating as well as carbon contamination present in the SEM-chamber (unnormalised wt.% concentrations were 8–11%). For analyses labelled 7–9, the carbon concentration was so high that most of it originated from the sample itself. Please note also that Li is missing, because it cannot be analysed using EDS.

Unnormalised wt.%

Label	C	O	F	Al	Si	P	S	Cl	V	Mn	Fe	Co	Ni	Cu	Total
1		27.02		0.47	0.07	0.2	0.18			16.89	1.06	18.26	18.15		82.3
2		30.9		0.6	0.09	0.09				55.37		0.93			87.98
3		33.71		0.56						56.35	0.65	1.09			92.36
4		31.41		0.67	0.08	0.07				56.35		1.03			89.61
5		59.66		34.88						0.17	0.39				95.11
6		27.67		2.51		0.22				1.06	1.42	8.23	44.08	1.45	86.64
7	45.62	29.93		0.31	0.17	13.17	0.1	0.17	0.72	0.65	25.46			2.13	118.93
8	41.97	28.37	2.89	0.23		16.26			0.26	0.41	28.3			0.59	119.29
9	82.25	7.94		0.16	15.12	1.65	0.12	0.39		0.47	3.98				112.74

at.%

Label	C	O	F	Al	Si	P	S	Cl	V	Mn	Fe	Co	Ni	Cu	Total
1		63.34		0.65	0.1	0.24	0.21			11.53	0.72	11.62	11.6		100
2		64.74		0.74	0.11	0.1				33.78		0.53			100
3		66.18		0.65						32.22	0.37	0.58			100
4		64.65		0.81	0.09	0.08				33.78		0.58			100
5		74.11		25.69						0.06	0.14				100
6		62.03		3.34		0.26				0.69	0.91	5.01	26.93	0.82	100
7	57.22	28.18		0.17	0.09	6.41	0.05	0.07	0.21	0.18	6.87			0.5	100
8	53.91	27.36	2.34	0.13		8.1			0.08	0.12	7.82			0.14	100
9	85.17	6.18		0.07	6.69	0.66	0.05	0.14		0.11	0.89				100

Table S5. Unnormalised wt.% and at.% results from the EDS analyses of points indicated in Fig. 3b. For explanation regarding the missing carbon in analysis area 11, the reader is referred to the caption of Table S4.

Unnormalised wt.%

Label	C	O	F	Al	Si	P	S	Cl	V	Mn	Fe	Co	Ni	Cu	Total
**10**	34.31	33.05		0.25	0.21	14.85			0.88	0.63	24.93			2.37	111.48
**11**		24.26	3.37	1.67		0.3				0.57	1.16	8.05	43.46	0.95	83.8
**12**	42.64	31.56	10.66	0.77	0.14	3.9	0.12	0.07	0.12	38.94	4.73	1.44	0.78	2.05	137.9

at.%

Label	C	O	F	Al	Si	P	S	Cl	V	Mn	Fe	Co	Ni	Cu	Total
**10**	48.16	34.83		0.16	0.13	8.08			0.29	0.19	7.53			0.63	100
**11**		56.41	6.59	2.3		0.36				0.39	0.78	5.08	27.54	0.56	100
**12**	49.9	27.73	7.89	0.4	0.07	1.77	0.05	0.03	0.03	9.96	1.19	0.34	0.19	0.45	100

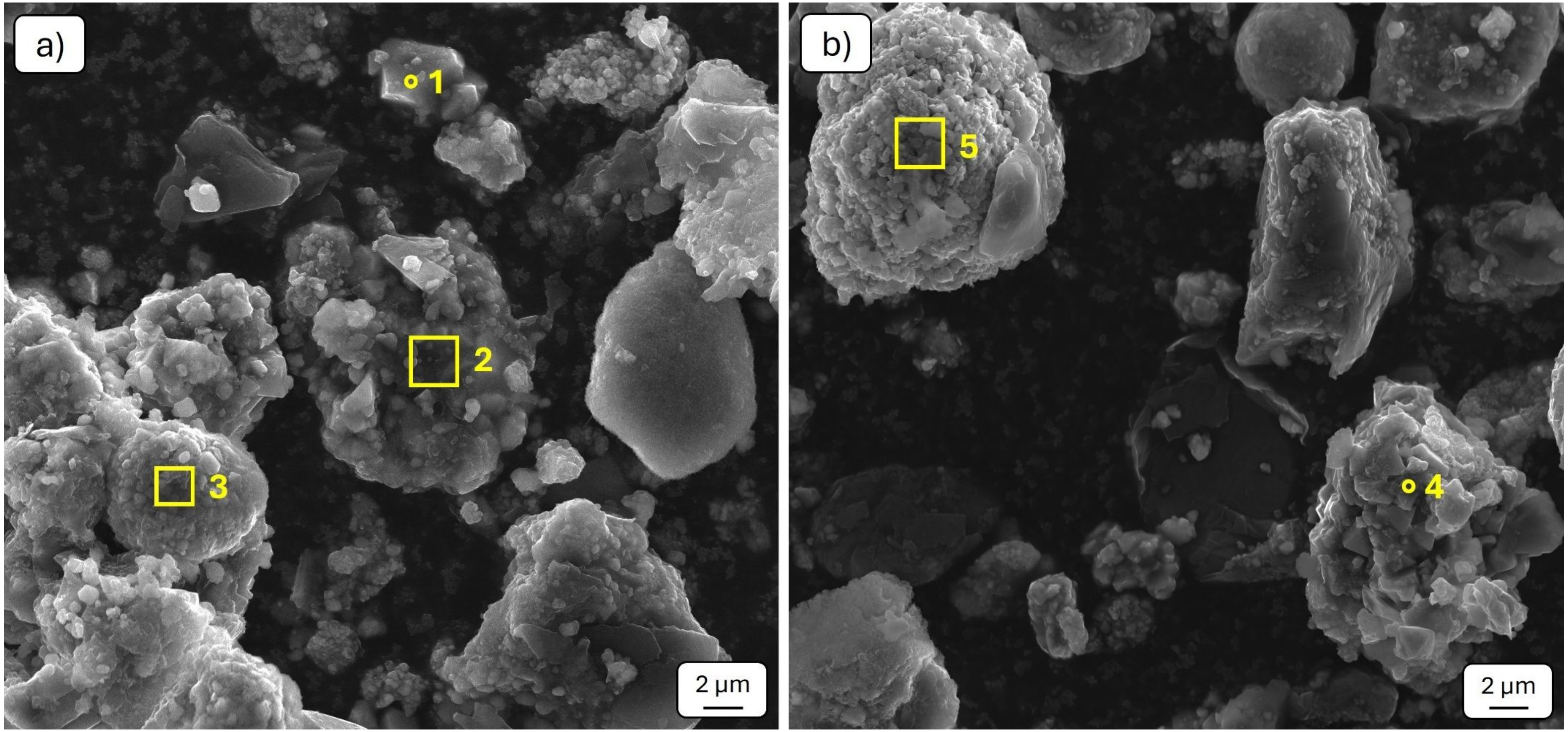

Figure S4. SEM-SE images a) and b) of <500 µm LFP-based black mass powder. Areas where qualitative EDS analyses were performed are marked with numbers 1–5. Areas 1, 2 and 4 are Mn oxide particles or their agglomerates. Area 3 is Ni-Co oxide particle and area 5 is an LFP particle agglomerate.

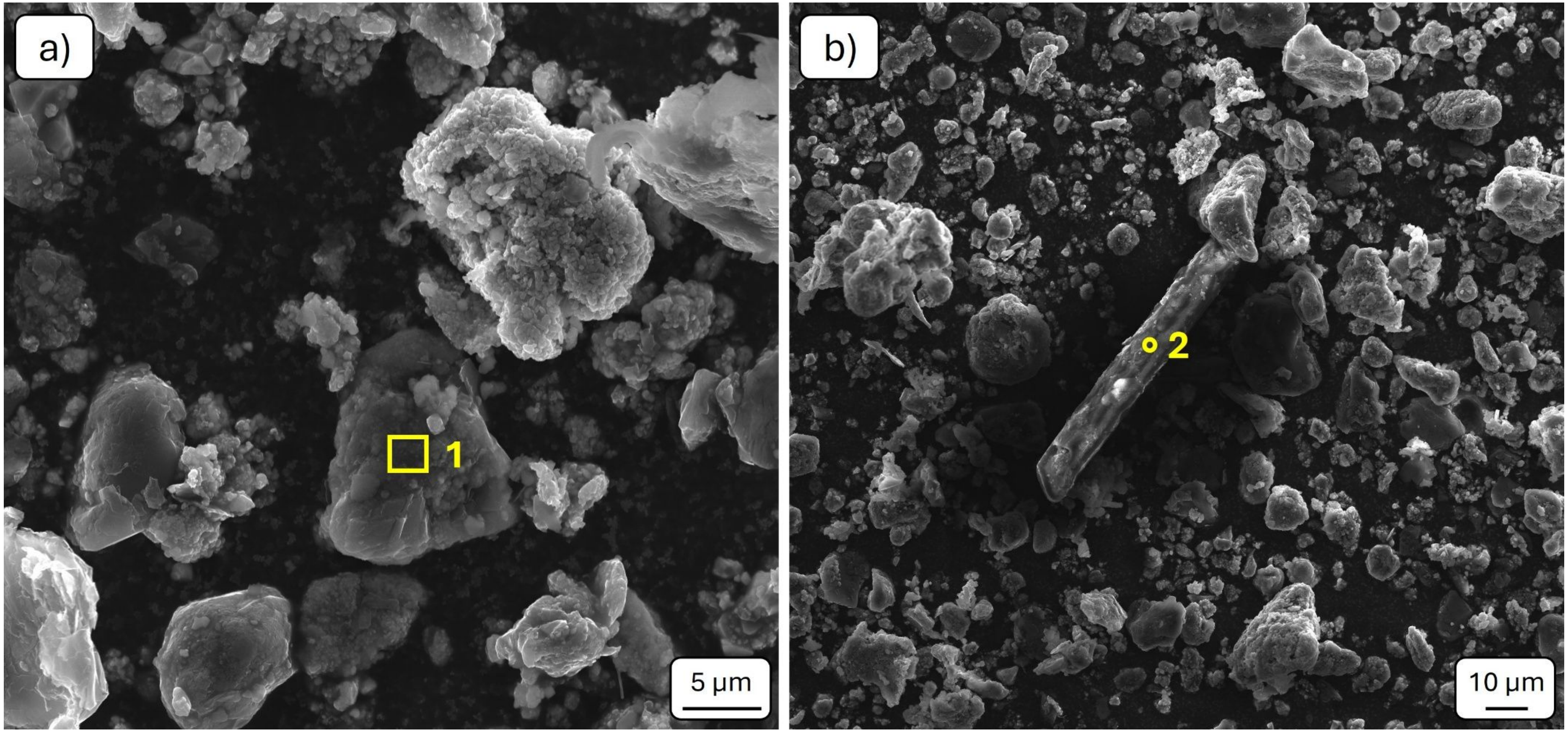

Figure S5. SEM-SE images a) and b) of <500 µm LFP-based black mass powder. Areas where qualitative EDS analyses were performed are marked with numbers 1–2. Both of these areas had high concentrations of Si, but area 1 had additionally high F and C contents, as well as low concentrations of P and Fe. The particle denoted by number 2 is shaped like a rod, and was composed of Si, Ca and Al oxides according to EDS data. In this particle, almost no C was detected.

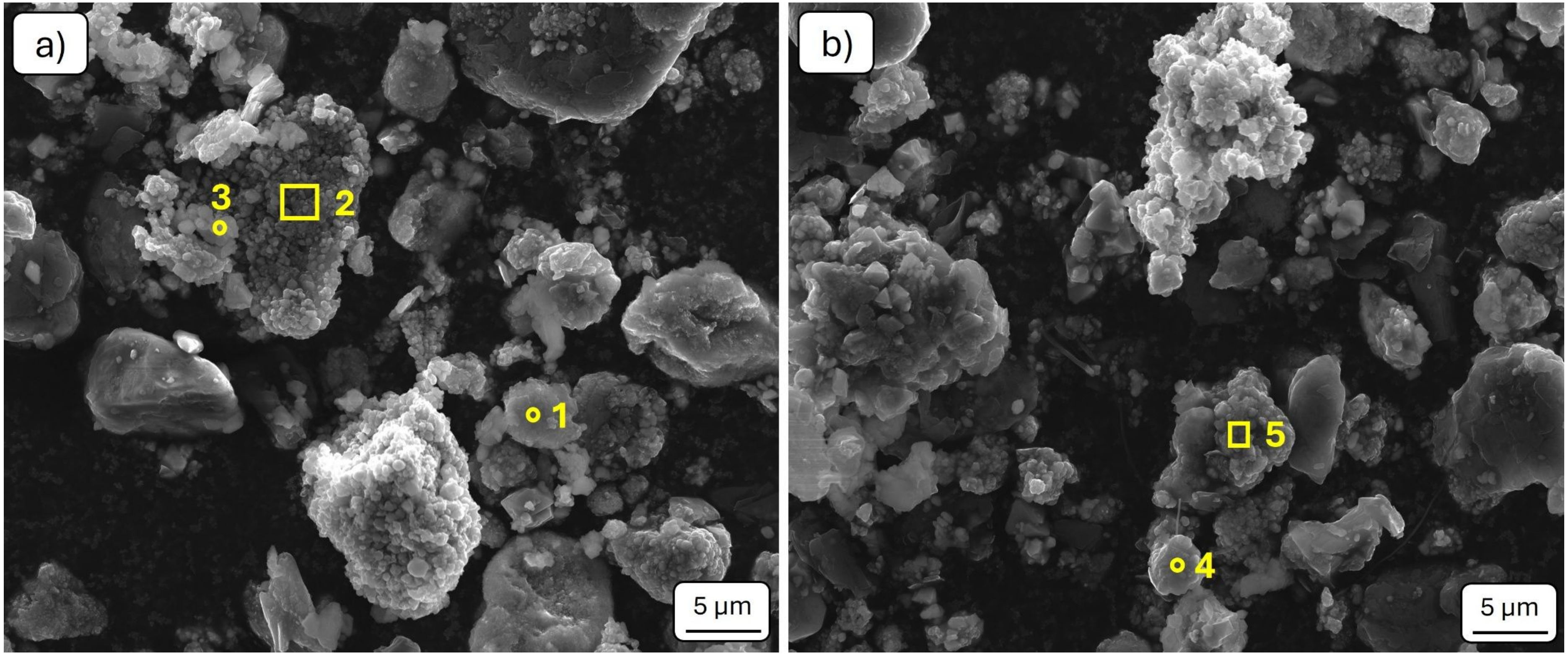

Figure S6. SEM-SE images a) and b) of <500 µm LFP-based black mass powder. Areas and points where qualitative EDS analyses were performed are marked with numbers 1–5. Points 1, 4 and 5 are LMFP particles with Fe:P atomic ratio around 1, and Mn amount 0.15–0.4 times the amount of Fe (in at.%). Area 2 is an LFP agglomerate, and the small particles right next to this agglomerate (point 3) are rich in Ti oxides.

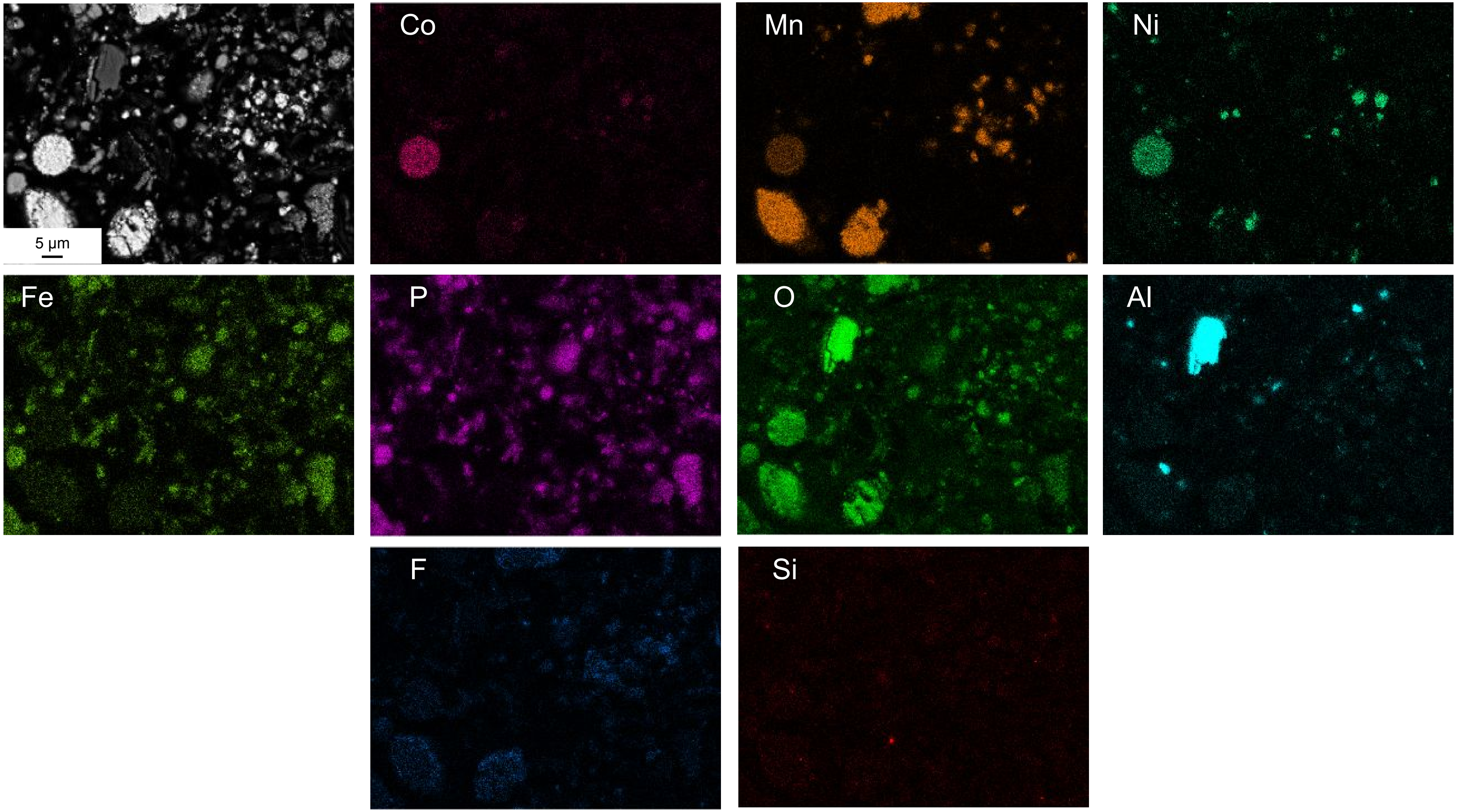

Figure S7. Elemental EDS mapping of <500 µm size fraction from a polished section (same area as shown in Fig. 3a). Mn is generally not found in the same particles as Fe and P. Only one of the particles containing Ni and Co also contained significant concentrations of Mn (the roundest particle towards the left side of the figure).

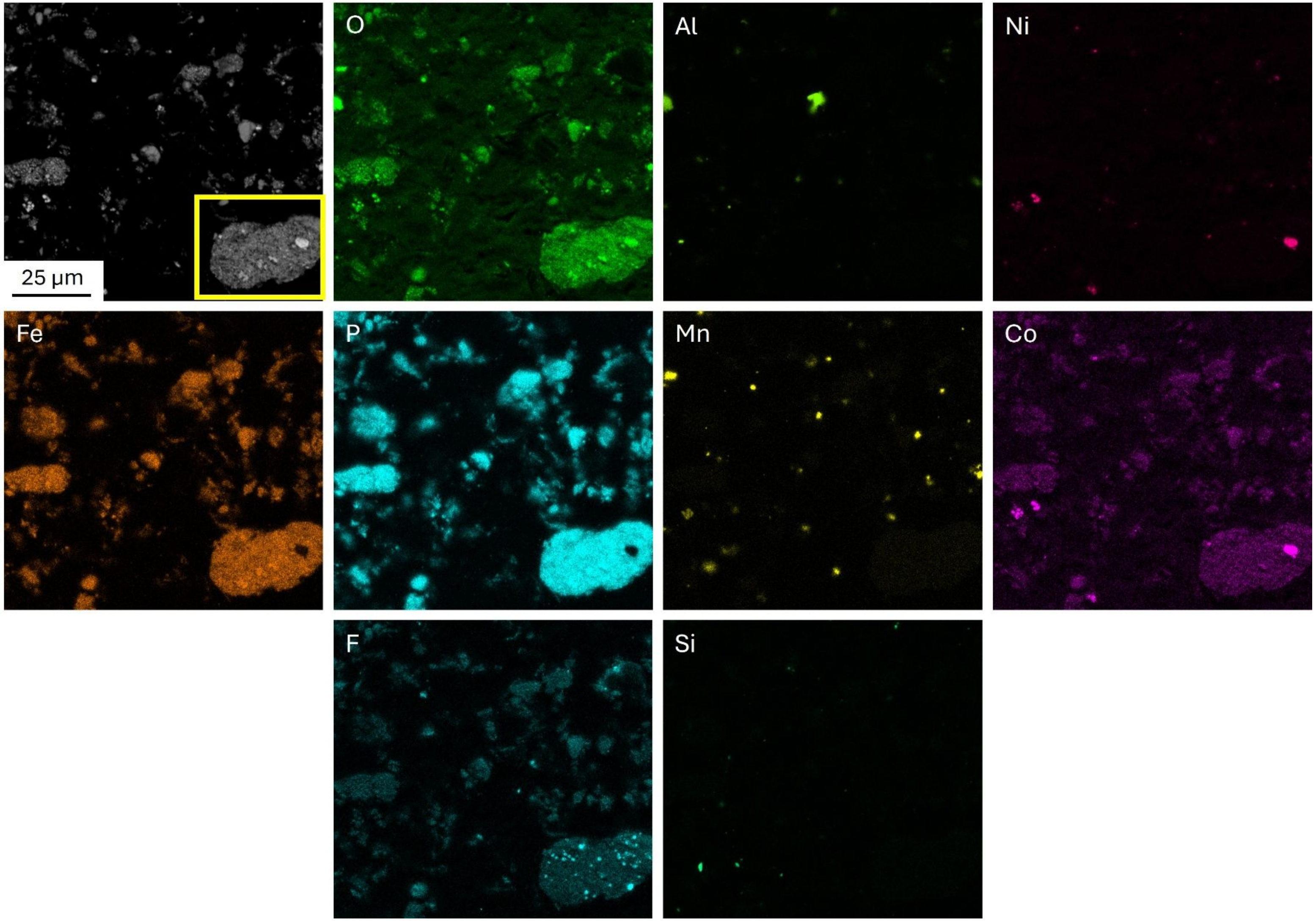

Figure S8. Elemental EDS mapping of another area in the polished section of <500 µm size fraction sample. Fe and P (LFP) containing particles clearly form the majority of the sample in this area. More detailed characterization of the larger particle highlighted with a yellow rectangle is presented in the next Figure S9 and Table S6.

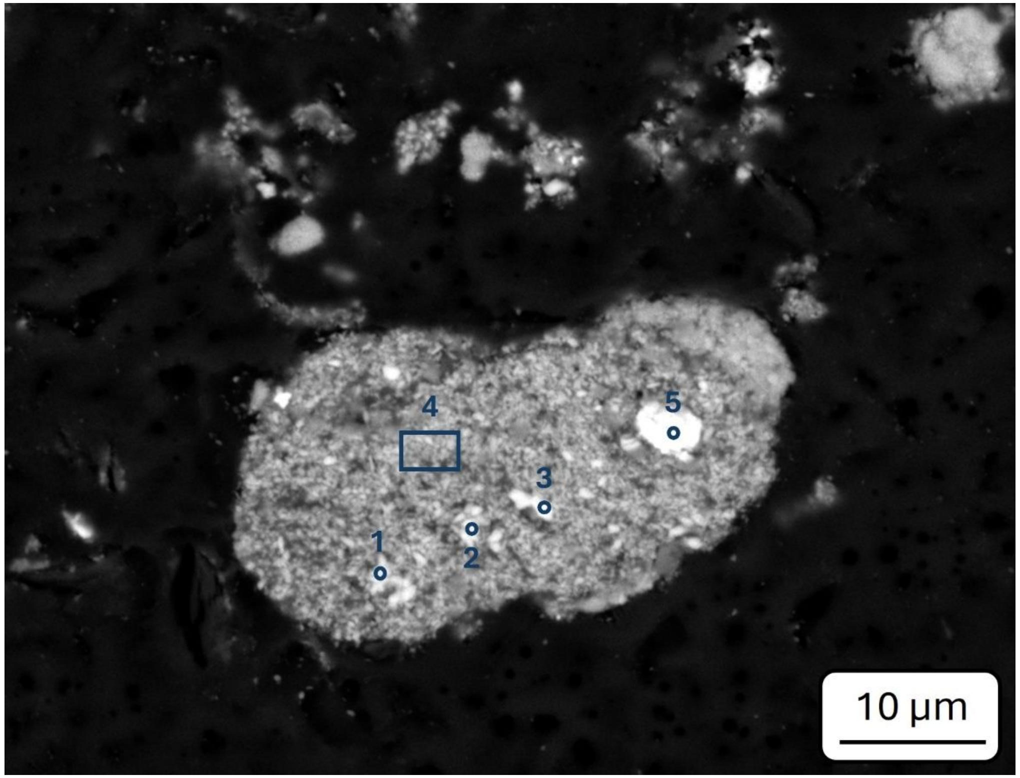

Figure S9. A close-up of the particle highlighted with the yellow rectangle in Figure S8. Points and area 1-5 indicate the location of the EDS analyses, shown in Table S6.

Table S6. Quantitative EDS results (at.%) of the areas indicated in Figure S9.

	at.%													
**Label**	**O**	**F**	**Al**	**Si**	**P**	**S**	**Ca**	**V**	**Mn**	**Fe**	**Co**	**Ni**	**Cu**	**Zr**	**Total**
1	57.51	0.72	0.1	0.13	20.14	0.24	0.07	0.01	0.43	19.28	0.42	0.12	0.64	0.2	100
2	65.45	0	0.06	0.16	16.94	0.19	0.02	0.01	0.33	15.95	0.41	0.11	0.25	0.13	100
3	64.61	0	0.05	0.11	17.15	0.16	0.04	0.02	0.31	16.74	0.4	0.05	0.15	0.2	100
4	57.28	2.17	0.09	0.22	20.33	0.3	0.04	0.01	0.54	17.24	0.38	0.13	1.04	0.22	100
5	59.61	4.19	1.92	0.15	0.59	0.04	0	0.01	0.41	1.17	5.11	26.06	0.73	0.01	100

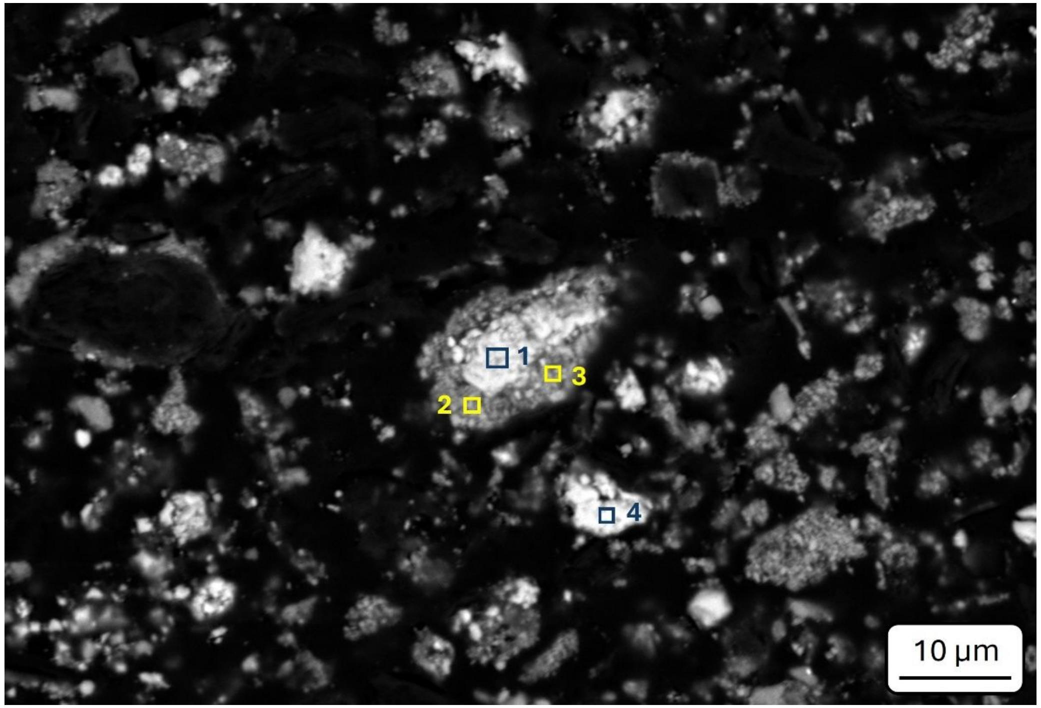

Figure S10. A close-up of the particle shown at the top of Fig. 4. The EDS analysis results from areas 1-4 are presented in Table S7.

Table S7. Quantitative EDS results (at.%) of areas 1-4 shown in Fig. S10.

	at.%														
Label	O	F	Al	Si	P	S	Cl	Ca	V	Mn	Fe	Co	Ni	Cu	Zr	Total
1	57.47	7.52	0.5	0.07	0.22	0.03	0	0.02	0.02	32.24	1.08	0.69	0.13	0	0.01	100
2	57.15	1.54	0.92	0.24	18.49	0.18	0.05	0.05	0.14	1.53	18.57	0.4	0.27	0.32	0.17	100
3	51.28	11.86	0.57	0.16	15.53	0.21	0	0.04	0.13	4.58	14.08	0.45	0.24	0.76	0.12	100
4	58.06	3.5	0.01	0.19	0.16	0.05	0.01	0	0.03	7.39	1.11	5.42	23.03	0.95	0.09	100

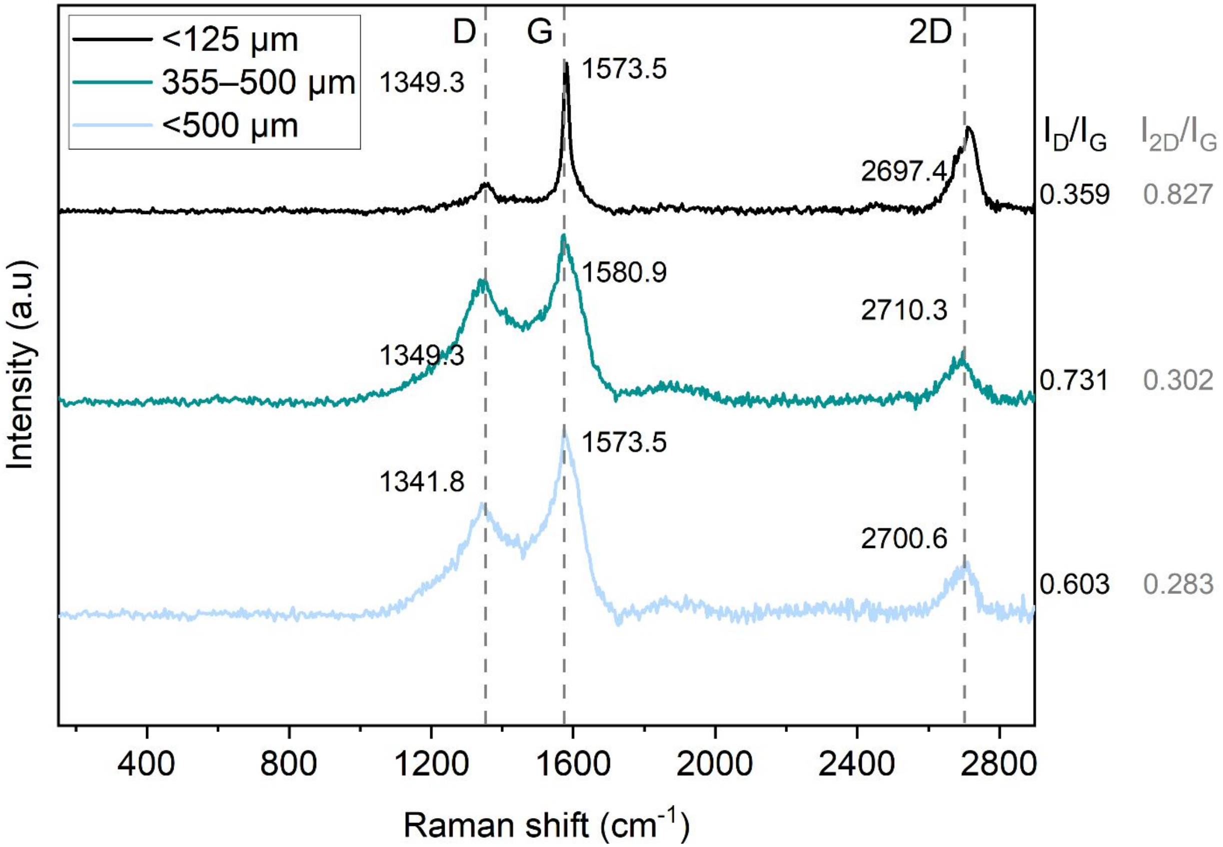

Figure S11. The results of Raman spectroscopy for <500, <125, and 355–500 μm fractions.
